# Nephroprotective Effects of Quercetin–Selenium Nanoparticles Against Glycerol-Induced AKI

**DOI:** 10.3390/ijms262412187

**Published:** 2025-12-18

**Authors:** Ahmed M. Ashour, Ali Khames, Khaled M. Alam-ElDein, Ahmed Hassan Ibrahim Faraag, Nievin Ahmed Mahran, Badriyah Aljazzaf, Rabia Alghazeer, Fatma Akmal, Marwa Ahmed Mahmoud, Mohamed H. A. Gadelmawla

**Affiliations:** 1 Department of Pharmacology and Toxicology, College of Pharmacy, Umm Al-Qura University, P.O. Box 13578, Makkah 21955, Saudi Arabia; 2 Department of Pharmacology and Toxicology, Faculty of Pharmacy, Sohag University, Sohag 82511, Egypt; 3 Molecular Biology and Biotechnology Department, School of Biotechnology, Badr University in Cairo, Cairo 11829, Egypt; 4 Botany and Microbiology Department, Faculty of Science, Helwan University, Cairo 11795, Egypt; 5 Department of Biochemistry, Faculty of Biotechnology, Sinai University, Kantara Branch, Ismailia 41636, Egypt; 6 Department of Food Sciences and Nutrition, College of Health Sciences, The Public Authority for Applied Education and Training, Kuwait City 13092, Kuwait; 7 Chemistry Department, Faculty of Science, University of Tripoli, Tripoli 50676, Libya; 8 Human Anatomy and Embryology Department, Faculty of Medicine, Zagazig University, Zagazig 44519, Egypt; 9 Department of Medical Physiology, Faculty of Medicine, Sohag University, Sohag 82511, Egypt; 10 Department of Life Sciences, Faculty of Biotechnology, Sinai University, Kantara Branch, Ismailia 41636, Egypt

**Keywords:** selenium, renal injury, quercetin, inflammation, oxidative stress, apoptosis

## Abstract

Acute kidney injury (AKI) is defined as a quick and often reversible decline in renal performance, as shown by elevated creatinine or reduced urine volume. AKI is a common illness, particularly among hospitalized cases, and can be observed in up to 7% of hospital admissions and 30% of ICU admissions. This study was designed to explore the nephroprotective potential of eco-synthesized quercetin–selenium nanoparticles (QUR-SeNPs) against experimentally glycerol-induced rhabdomyolysis leading to AKI. Forty healthy adult male albino rats were employed in the experiment. Animals were randomly distributed equally into five groups: Control: orally administered with normal saline solution. GLY: orally administered with normal saline (0.9% NaCl) for 15 consecutive days, at day 14, animals of this group received a single dose of intramuscular (im.) injection of 50% glycerol (GLY) (10 mg/kg/day). GLY and quercetin (GLY&QUR): orally administered with quercetin daily for 15 days (50 mg/kg/day), at day 14, animals of this group received a single dose of im. injection of 50% glycerol (10 mg/kg/day). GLY&Na_2_SeO_3_: orally administered with sodium selenite daily for 15 days (0.5 mg/kg/day), at day 14, animals of this group received a single dose of im. injection of 50% glycerol (10 mg/kg/day). GLY&QUR-SeNPs: orally administered with selenium nanoparticles synthesized using quercetin daily for 15 days (0.5 mg/kg/day), at day 14, animals of this group received a single dose of im. injection of 50% glycerol (10 mg/kg/day). Oxidative stress, inflammatory, and apoptotic markers, in addition to histopathological, gene expression, and immunohistochemical analysis, were assessed for all groups. The results demonstrated that QUR-SeNPs effectively ameliorated renal functional, biochemical, and molecular disturbances through their synergistic antioxidant, anti-inflammatory, and anti-apoptotic potential, surpassing the effects of either quercetin or selenium alone. Biosynthesized selenium nanoparticles using QUR-SeNPs demonstrated remarkable nephroprotective activity by normalizing renal biomarkers, restoring antioxidant capacity, inhibiting inflammatory cytokines, and preventing apoptotic damage. The nanoparticle formulation exhibited superior efficacy to either QUR or Se alone, highlighting the synergistic interplay between selenium and quercetin through enhanced bioavailability, redox stability, and molecular targeting.

## 1. Introduction

Acute kidney injury (AKI) continues to be a major concern in both clinical and experimental toxicology due to its intricate pathophysiology and the significant public health burden it imposes. The kidneys, essential for maintaining homeostasis, are highly susceptible to toxic agents, which can impair their function and lead to various pathologies, including AKI. Intramuscular glycerol injection induces rhabdomyolysis, causing acute skeletal muscle breakdown, resulting in the release of myoglobin and other nephrotoxic metabolites. These factors precipitate renal vasoconstriction, oxidative stress, and tubular obstruction, collectively causing acute kidney injury [1,2]. The mechanisms underlying AKI are multifactorial, involving oxidative stress (OS), inflammation, apoptosis, and fibrosis, all of which contribute to the continuous deterioration of kidney function [3]. These pathological insults disrupt cellular integrity, alter renal hemodynamics, and cause dysfunction in the glomerular and tubular structures, resulting in impaired renal performance [4]. Despite extensive research, management approaches aimed at preventing or mitigating renal damage remain insufficient, underscoring the need for further investigation into novel treatment options.

Existing research has highlighted several triggers of nephrotoxicity, including the use of certain pharmacological agents (such as cisplatin and gentamicin), exposure to environmental pollutants, and underlying conditions like diabetes and hypertension [5,6]. OS is widely recognized as a pivotal factor in renal damage, with reactive oxygen species (ROS) playing a central role in cellular injury. The excessive generation of ROS leads to lipid peroxidation, protein modification, and DNA damage, exacerbating the inflammatory response within the kidney tissue [7]. Additionally, pro-inflammatory cytokines are remarkably increased following toxic injury, activating pro-fibrotic pathways that promote tissue scarring and chronic renal dysfunction [8,9]. Apoptotic pathways, marked by a rise in pro-apoptotic proteins such as Bax and caspase-3, alongside a reduction in the anti-apoptotic protein Bcl-2, contribute significantly to renal cell death and functional impairment [9].

Although the roles of OS and inflammation in kidney toxicity are well-established, the precise molecular mechanisms that govern these processes are not yet fully understood. Furthermore, there remains a notable absence of effective pharmacological interventions capable of addressing the multifaceted nature of renal injury [10]. Current treatments often focus on symptom management rather than reversing the underlying damage, and there is limited success in preventing the progression to kidney failure. As such, there is a pressing need for novel, multi-targeted therapeutic modules that can comprehensively address the various facets of renal injury and promote recovery [11].

Quercetin, a naturally occurring flavonoid found abundantly in fruits, vegetables, and grains, has gained considerable attention for its potential therapeutic benefits, particularly in the context of renal protection [5]. This compound has robust antioxidant, anti-inflammatory, and anti-apoptotic features, positioning it as a promising candidate for mitigating AKI [12]. Numerous studies have demonstrated that quercetin can alleviate OS by scavenging free radicals and promoting the activity of endogenous antioxidant enzymes. In addition to its antioxidant effects, quercetin modulates pro-inflammatory cytokines and inhibits apoptotic signaling, further supporting its therapeutic potential in mitigating kidney damage [13,14].

A vital trace element, selenium is necessary for preserving redox equilibrium and protecting against cellular damage caused by OS. It is incorporated into selenoproteins, such as glutathione peroxidases and thioredoxin reductases, which are pivotal in neutralizing ROS and maintaining cellular integrity [15]. In renal tissues, selenium’s antioxidant properties help reduce oxidative damage and inflammation, both key contributors to nephrotoxicity [16]. Additionally, selenium has been shown to modulate inflammatory pathways and support cellular repair mechanisms, further enhancing its protective role against kidney injury [17].

In light of these observations, we hypothesized that green-synthesized selenium nanoparticles biosynthesized using quercetin would exert superior nephroprotective activity compared with quercetin or selenium administered alone. This expectation is based on the proposed synergistic interaction between quercetin’s potent radical-scavenging and anti-inflammatory actions and selenium’s critical role in selenoprotein-mediated redox homeostasis. Nanoparticle formation is further anticipated to enhance bioavailability, protect quercetin from rapid degradation, and facilitate improved renal cellular uptake. These combined advantages represent a novel therapeutic strategy for augmenting the protective potential of both agents in glycerol-induced rhabdomyolysis-associated acute kidney injury. This study was designed to explore the nephroprotective potential of eco-synthesized quercetin–selenium nanoparticles (QUR-SeNPs) against experimentally glycerol-induced rhabdomyolysis leading to acute kidney injury (AKI).

## 2. Results

### 2.1. Molecular Docking Analysis

**Figure 1:**
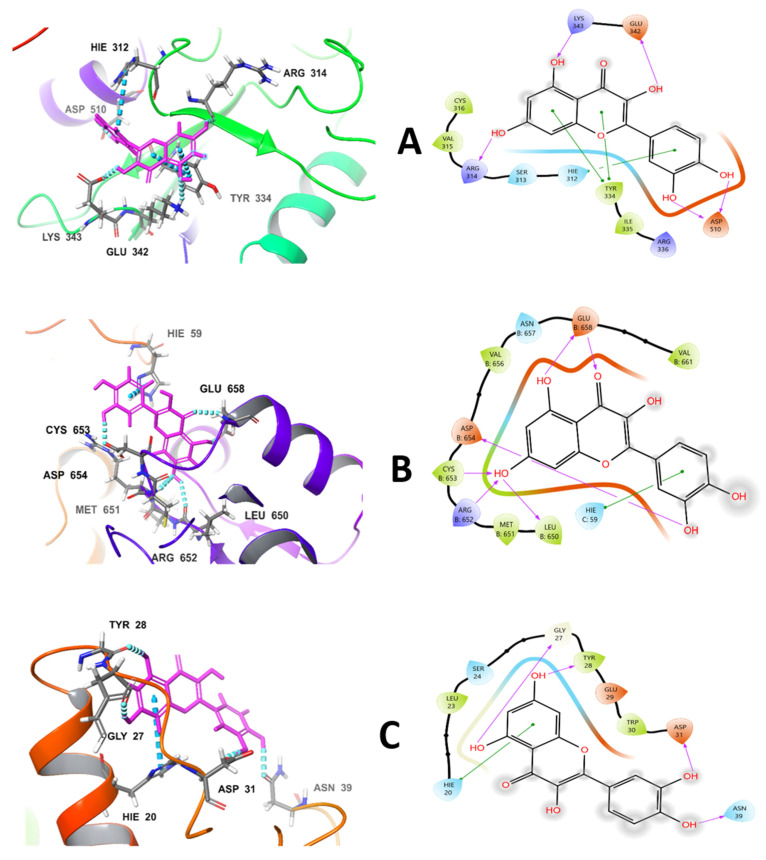
Interaction diagrams and 3D representations of quercetin docked into the active sites of (**A**) PTPN5, (**B**) DQC ubiquitin ligase, and (**C**) BCL-2 isoform 1.

The molecular docking analysis revealed a discernible trend in quercetin’s predicted binding affinities across the three target proteins. Quercetin demonstrated the most favorable binding interaction with the Dimerization Quality Control (DQC) ubiquitin ligase, achieving a Glide Docking score of −6.34 kcal mol^−1^ (Table 1 and Figure 1), complemented by an Emodel value of −49.32 kcal mol^−1^ (Table 1). This suggests a strong predicted affinity for the DQC catalytic site. A slightly weaker, yet still notable, binding was predicted for BCL-2 isoform 1, with a Docking score of −5.41 kcal mol^−1^ (Table 1) and an Emodel of −39.13 kcal mol^−1^ (Table 1). In contrast, the interaction with human PTPN5 yielded a Docking score of −5.35 kcal mol^−1^ (Table 1), indicating a comparable binding affinity to BCL-2, but it was accompanied by the most negative Emodel of −53.40 kcal mol^−1^ (Table 1). These results collectively propose a modest in silico selectivity of quercetin for the DQC ubiquitin ligase binding site over the PTPN5 and BCL-2 proteins. However, it is crucial to acknowledge that the absence of replicates across docking runs or the inclusion of additional reference ligands for comparative analysis preclude robust statistical validation of this observed selectivity. Further experimental and computational studies would be necessary to confirm these preliminary findings and to fully elucidate the binding mechanisms.

The binding affinities observed for quercetin across the three proteins are directly attributable to the specific types and strengths of interactions formed within their active sites. The DQC ubiquitin ligase demonstrates the highest affinity, characterized by a dense network of four direct hydrogen bonds and substantial π/alkyl contacts, which comprehensively explains the favorable −6.34 kcal mol^−1^ Docking score. BCL-2 exhibits moderate affinity (−5.41 kcal mol^−1^). Based on the provided data, quercetin’s interactions with three different protein targets reveal distinct binding modes and affinities. With BCL-2 (PDB-ID: 1G5M), it establishes a moderate affinity through a π–π-stack with Tyr-28 and hydrogen bonds with Asp-31, Gly-27, and Asn-39, supplemented by Van der Waals forces with His-20. These interactions occur within the BH3-mimetic groove. Lastly, PTPN5, despite registering as the most negative Emodel, shows a slightly weaker gscore of −5.35 kcal mol^−1^. This pose is nevertheless well-anchored by two critical arginine grips, which compensate for a comparatively smaller hydrophobic surface area, indicating a robust, albeit less energetically favorable, interaction (Table 2 and Figure 1). Its binding to PTPN5 (PDB-ID: 2BIJ) is characterized by a slightly weaker affinity. This interaction is anchored by hydrogen bonds with Arg-314 and His-312, a bifurcated hydrogen bond with Glu-342 and Asp-510, a π-stack with Tyr-334, and an electrostatic interaction with Lys-343. The presence of two arginine grips helps to compensate for a smaller hydrophobic surface area. Quercetin demonstrates the highest affinity for DQC (PDB-ID: 6WCQ). This strong interaction is attributed to a dense network of interactions, including the chelation of a structural water molecule by Asp-654, a hydrogen bond with His-59, a salt-bridge-like interaction with Arg-652, and another hydrogen bond with Met-651. The binding is further stabilized by Van der Waals contacts with Leu-650, Val-656, and Met-651, as well as robust π/alkyl contacts.

### 2.2. Characterization of Selenium Nanoparticles Biosynthesized Using Quercetin Particle Size and Zeta Potential

**Figure 2:**
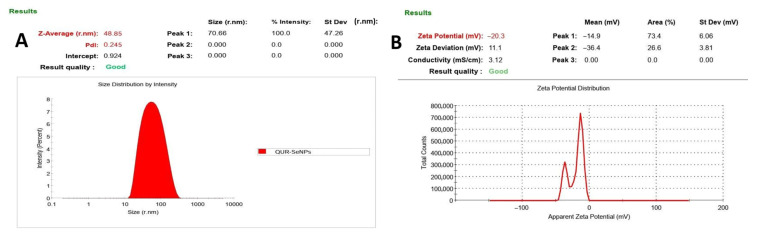
(**A**) Particle Size through DLS and (**B**) zeta potential analyses of quercetin-mediated selenium nanoparticles (QUR-SeNPs) showing nanoscale particle size distribution and a negative surface charge.

Dynamic light scattering (DLS) analysis revealed that the quercetin-mediated selenium nanoparticles (QUR-SeNPs) exhibited a Z-average hydrodynamic diameter of 48.85 nm with a polydispersity index (PDI) of 0.245, indicating a narrow and homogeneous particle size distribution. A single sharp peak centered at 70.66 nm (100% intensity) confirmed the formation of uniformly dispersed nanoparticles with minimal aggregation. The zeta potential measurement showed a mean surface charge of −20.3 mV, with two peaks at −14.9 mV (73.4%) and −36.4 mV (26.6%), reflecting moderate negative charge sufficient to prevent particle agglomeration through electrostatic repulsion. The measured conductivity (3.12 mS/cm) and zeta deviation (11.1 mV) further supported the stability and reliability of the colloidal suspension, as shown in Figure 2.

### 2.3. Transmission Electron Microscopy Analysis

**Figure 3:**
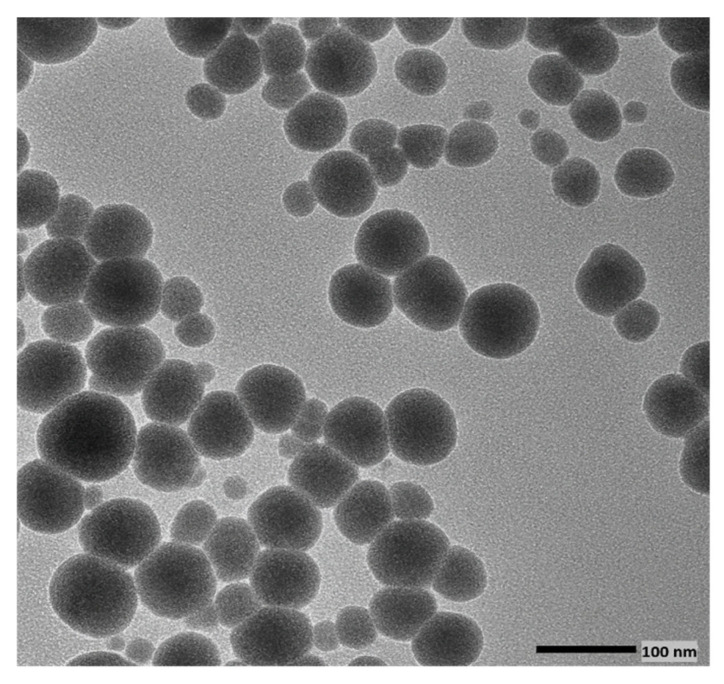
Transmission Electron Microscopy (TEM) image of quercetin-mediated selenium nanoparticles (QUR-SeNPs) showing uniformly distributed, spherical, and well-dispersed particles with sizes below 100 nm.

TEM provided detailed insight into the morphology and size distribution of the quercetin-mediated selenium nanoparticles (QUR-SeNPs). As shown in Figure 3, the nanoparticles appeared predominantly spherical, well-dispersed, and uniform in shape, with smooth boundaries and minimal aggregation. The particles exhibited a narrow size distribution with diameters typically below 100 nm, indicating the successful synthesis of nanoscale selenium structures. The absence of significant clustering suggests effective stabilization by quercetin molecules, which acted as both reducing and capping agents during nanoparticle formation. The clear contrast and defined edges observed in the TEM micrograph further confirm the homogeneity and crystalline nature of the nanoparticles, consistent with the formation of stable, monodisperse SeNPs suitable for biological applications.

### 2.4. Fourier Transform Infrared (FTIR) Analysis

**Figure 4:**
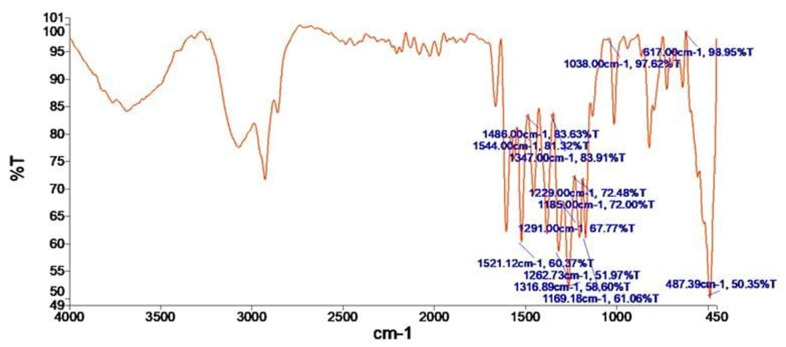
FTIR spectrum of quercetin-mediated selenium nanoparticles (QUR-SeNPs) showing characteristic absorption bands of functional groups involved in the reduction and stabilization of selenium nanoparticles by quercetin.

FTIR spectrum of quercetin-mediated selenium nanoparticles (QUR-SeNPs) exhibited distinct absorption bands confirming the successful formation and surface functionalization of the nanoparticles. Prominent peaks were observed at 1544, 1521, and 1486 cm^−1^, corresponding to aromatic C=C stretching vibrations of the quercetin phenolic rings. Bands appearing at 1347, 1317, and 1291 cm^−1^ were attributed to C–O and C–H bending modes, whereas absorptions at 1263 and 1229 cm^−1^ reflected C–O–C stretching of ether or hydroxyl groups. Additional peaks at 1185 and 1169 cm^−1^ correspond to C–O stretching of phenolic compounds, while the intense band near 1038 cm^−1^ was assigned to C–O deformation and possible Se–O bond formation. The low-frequency region revealed bands at 617 and 487 cm^−1^, characteristic of Se–O and Se–Se vibrational modes, respectively, confirming selenium incorporation into the nanoparticle matrix as shown in Figure 4.

### 2.5. Assessment of Kidney Weight and Relative Kidney Weight

**Figure 5:**
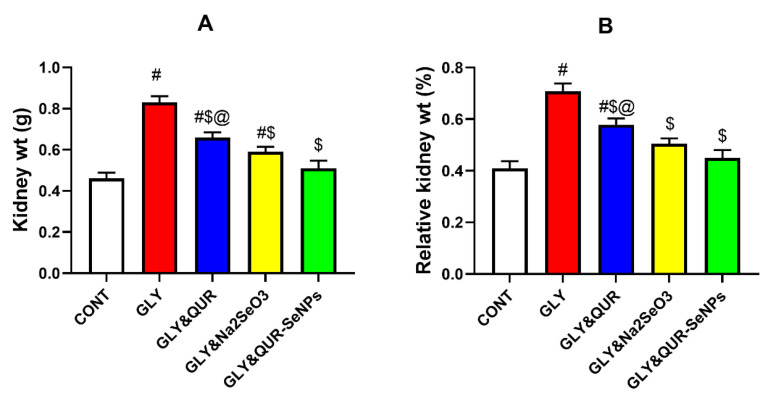
Effect of QUR, Na_2_SeO_3_, and QUR-SeNPs on (**A**) kidney weight and (**B**) relative kidney weight (%) in glycerol-induced AKI in rats. Data are expressed as mean ± SEM (*n* = 8 per group). Statistical annotations: # vs. CONT; $ vs. GLY; @ vs. GLY&QUR-SeNPs (*p* < 0.05).

As shown in Figure 5, glycerol injection (GLY) produced a significant increase in both (A) kidney weight and (B) relative kidney weight, with elevations of 80.43% and 72.68% compared with the control group (CONT). Pre-treatment with quercetin (GLY&QUR) conferred partial improvement, reducing kidney weight and relative kidney weight compared with GLY by 20.48% and 18.36%, respectively. However, values remained markedly above CONT, by 43.48% and 40.98%, indicating incomplete protection. More pronounced effects were observed with sodium selenite (GLY&Na_2_SeO_3_), which lowered kidney weight and relative kidney weight relative to GLY by 28.92% and 28.53%, though elevations above CONT persisted at 28.26% and 23.41%.

Remarkably, animals pretreated with selenium nanoparticles biosynthesized using quercetin (GLY&QUR-SeNPs) exhibited the most robust renoprotection, with kidney weight and relative kidney weight showing only minor elevations compared with CONT (10.87% and 9.76%). Against GLY, these changes represented substantial reductions of 38.55% and 36.44%. Furthermore, values in the GLY&QUR-SeNPs group were consistently lower than those in the GLY&QUR group by 29.41% and 28.44%, confirming the superior efficacy of the nanoparticle formulation.

### 2.6. Assessment of Skeletal Muscle Health

**Figure 6:**
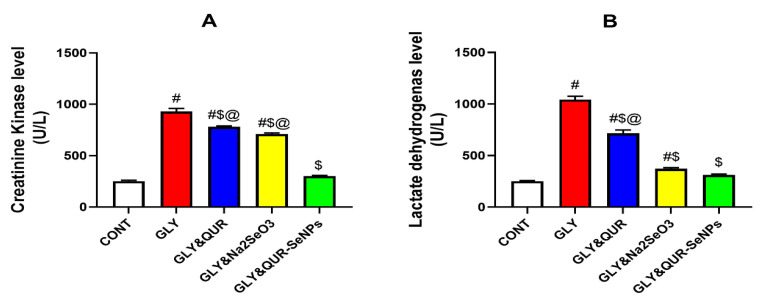
Effect of QUR, Na_2_SeO_3_, and QUR-SeNPs on (**A**) serum creatine kinase (CK) and (**B**) lactate dehydrogenase (LDH) in glycerol-induced AKI in rats. Data are expressed as mean ± SEM (*n* = 8 per group). Statistical annotations: # vs. CONT; $ vs. GLY; @ vs. GLY&QUR-SeNPs (*p* < 0.05).

As represented in Figure 6, the results demonstrated remarkable variances in serum CK (A) and LDH (B) activities among the experimental groups. Glycerol administration (GLY) markedly elevated CK +268.54% and LDH +313.93% compared with the control (CONT), confirming induction of renal and tissue injury.

Quercetin pre-treatment (GLY&QUR) partially attenuated these elevations, reducing CK and LDH versus GLY by −16.11% and −31.46%, respectively, yet remaining higher than CONT by +209.17% and +183.73%. Sodium selenite supplementation (GLY&Na_2_SeO_3_) exerted stronger protection, lowering CK by −23.62% and LDH −64.22% relative to GLY, though still above CONT by +181.50% and +48.13%. On the other hand, induction of selenium nanoparticles biosynthesized using quercetin (GLY&QUR-SeNPs) restored CK and LDH activities toward normal values, showing only +19.58% and +23.92% changes versus CONT, and profound reductions relative to GLY of −67.55% and −70.06%.

### 2.7. Assessment of Renal Function Biomarkers

**Figure 7:**
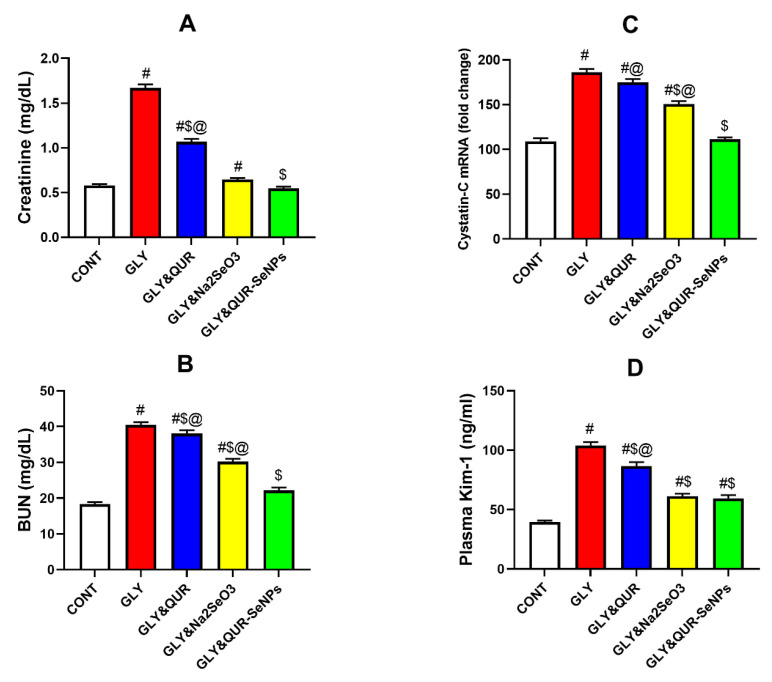
Effect of QUR, Na_2_SeO_3_, and QUR-SeNPs on renal function biomarkers: (**A**) serum creatinine, (**B**) blood urea nitrogen (BUN), (**C**) *cystatin-C*, and (**D**) KIM-1 in glycerol-induced AKI in rats. Data are presented as mean ± SEM (*n* = 8 per group). Statistical annotations: # vs. CONT; $ vs. GLY; @ vs. GLY&QUR-SeNPs (*p* < 0.05).

As shown in Figure 7, glycerol administration (GLY) induced pronounced renal impairment, on serum creatinine (A), BUN (B), homogenate of *cystatin-C* (C), and plasma kim-1 (D) reflected by significant increases as follows: +187.93%, +120.76%, +70.64%, and 163.50%. Respectively, compared with the control CONT.

The group of rats orally pre-administrated with quercetin (GLY&QUR) conferred partial protection, reducing these elevations versus GLY by −36.05%, −5.88%, −6.04%, and −16.65% though values remained higher than CONT by +84.13%, +107.77%, +60.33%, and +119.61%. Sodium selenite supplementation (GLY&Na_2_SeO_3_) offered stronger nephroprotection, with decreases relative to GLY of −61.32%, −25.29%, −19.02%, and −40.97% but the elevations above CONT were +11.38%, +64.92%, +38.18%, and +55.53%.

Remarkably, selenium nanoparticles synthesized with quercetin (GLY&QUR-SeNPs) achieved near normalization of renal markers, showing minimal changes versus CONT of −5.86%, +20.90%, +1.9%, and +50.93% and substantial reductions compared with GLY of −67.31%, −45.23%, −40.26%, and 42.72%.

### 2.8. Investigation of Oxidant/Antioxidant Biomarkers

**Figure 8:**
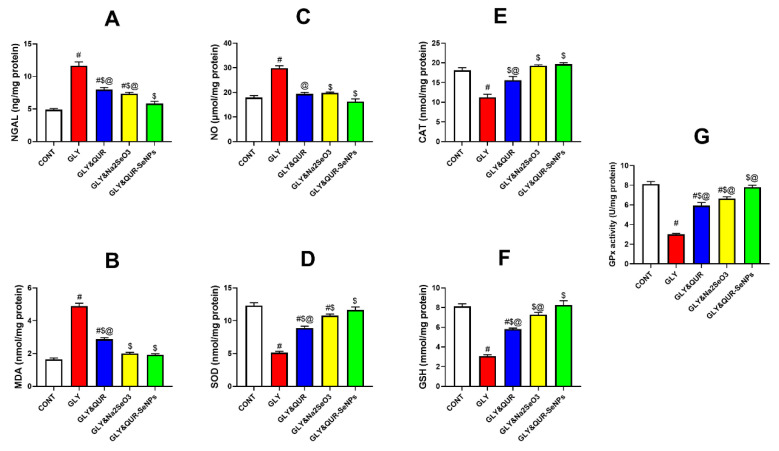
Effect of QUR, Na_2_SeO_3_, and QUR-SeNPs on OS and antioxidant biomarkers: (**A**) NGAL, (**B**) malondialdehyde (MDA), (**C**) nitric oxide (NO), (**D**) catalase (CAT), (**E**) superoxide dismutase (SOD), (**F**) reduced glutathione (GSH), and (**G**) glutathione peroxidase (GPx) in glycerol-induced AKI in rats. Data are expressed as mean ± SEM (*n* = 8 per group). Statistical annotations: # vs. CONT; $ vs. GLY; @ vs. GLY&QUR-SeNPs (*p* < 0.05).

As depicted in Figure 8, glycerol administration (GLY) elicited profound OS, evidenced by marked elevations in (A) NGAL (137.88%), (B) MDA (198.05%), and (C) NO (67.02%), concomitant with substantial declines in the antioxidant defense parameters (D) SOD (−58.33%), (E) CAT (−37.88%), (F) GSH (−62.37%), and (G) GPx (−63.06%) relative to the control (CONT). These alterations clearly verify the successful induction of oxidative renal injury.

Pre-treatment with quercetin (GLY&QUR) conferred partial renoprotection, attenuating NGAL, MDA, and NO levels relative to GLY by 31.35%, 41.37%, and 35.09%, respectively, while simultaneously enhancing antioxidant capacity, as reflected by increases in SOD (73.10%), CAT (39.02%), GSH (89.86%), and GPx (97.81%). Nonetheless, values remained significantly different from CONT, indicating incomplete restoration. While oral induction with sodium selenite (GLY&Na_2_SeO_3_) produced more pronounced protective effects, yielding reductions in NGAL, MDA, and NO by 36.78%, 59.08%, and 33.59%, respectively, together with substantial elevations in SOD (109.67%), CAT (71.40%), GSH (138.39%), and GPx (121.02%) compared with GLY.

Most notably, animals pretreated with selenium nanoparticles biosynthesized using quercetin (GLY&QUR-SeNPs) exhibited the most potent antioxidant response. Compared with CONT, these animals demonstrated nearly normalized biomarker levels: NGAL (19.28%), MDA (17.05%), NO (−8.91%), SOD (−5.43%), CAT (8.87%), GSH (1.67%), and GPx (−3.82%). Relative to GLY, these changes corresponded to robust corrections of 49.85%, 60.73%, 45.47%, 126.98%, 75.26%, 170.18%, and 160.36%, respectively. These results show the superior antioxidant potential of QUR-SeNPs in restoring redox balance to normal state.

### 2.9. Investigation of Inflammatory Biomarkers Assessment

**Figure 9:**
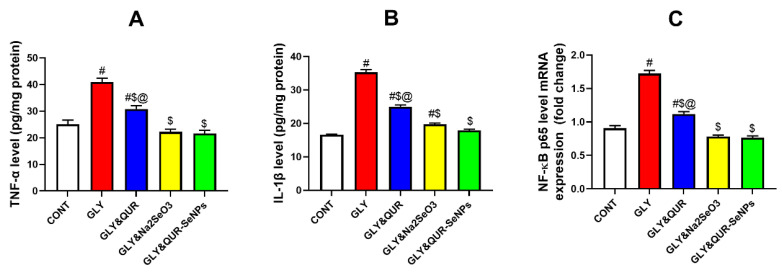
Effect of QUR, Na_2_SeO_3_, and QUR-SeNPs on inflammatory biomarkers: (**A**) tumor necrosis factor-α (TNF-α), (**B**) interleukin-1β (IL-1β), and (**C**) nuclear factor kappa B (*NF-κB*) in glycerol-induced AKI in rats. Data are presented as mean ± SEM (*n* = 8 per group). Statistical annotations: # vs. CONT; $ vs. GLY; @ vs. GLY&QUR-SeNPs (*p* < 0.05).

As represented in Figure 9, the results demonstrated remarkable variances (*p* < 0.05) in pro-inflammatory markers among the experimental groups. Glycerol administration (GLY) markedly elevated TNF +63.45%, IL-1 +112.69%, and *NF-κB* +90.28% compared with the control (CONT), confirming a strong inflammatory response.

Significantly reducing TNF (A), IL-1 (B), and NF-κB (C) versus GLY −24.69%, −29.10%, and −34.96%, respectively, by oral administration of quercetin as pre-treatment (GLY&QUR) provided partial attenuation, although the levels remained higher than CONT by +23.10%, +50.80%, and +23.76%. Additionally, sodium selenite (GLY&Na_2_SeO_3_) exerted stronger anti-inflammatory effects, significantly lowering TNF (−45.83%), IL-1 (−44.13%), and *NF-κB* (−54.59%) relative to GLY, approaching CONT values of −11.46%, +18.83%, and −13.59%.

The group of rats induced by selenium nanoparticles biosynthesized using quercetin (GLY&QUR-SeNPs) showed the most profound suppression, nearly normalizing inflammatory markers with only −13.68%, +8.10%, and −15.36% differences from CONT for TNF, IL-1, and *NF-κB*, respectively, with shown reductions against GLY of −47.19%, −49.18%, and −55.52%.

### 2.10. Assessment of Apoptotic/Anti-Apoptotic Biomarkers

**Figure 10:**
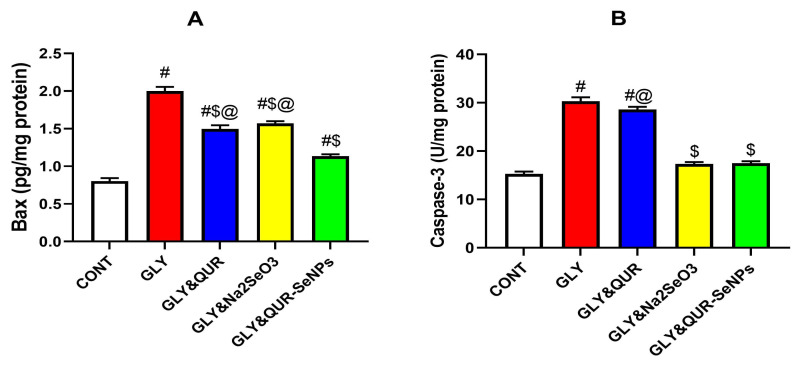
Effect of QUR, Na_2_SeO_3_, and QUR-SeNPs on apoptotic markers: (**A**) Bax expression and (**B**) caspase-3 activity in glycerol-induced AKI in rats. Data are expressed as mean ± SEM (*n* = 8 per group). Statistical annotations: # vs. CONT; $ vs. GLY; @ vs. GLY&QUR-SeNPs (*p* < 0.05).

As shown in Figure 10, glycerol administration (GLY) elicited a profound activation of the intrinsic apoptotic pathway. This was evidenced by a significant 148.5% elevation in Bax expression relative to the control (CONT), accompanied by a 97.97% increase in caspase-3 activity, indicating extensive pro-apoptotic signaling. Quercetin pre-treatment (GLY&QUR) partially attenuated these alterations, reducing Bax levels by 25.1% and caspase-3 activity by 5.56% compared with GLY; however, both markers remained markedly elevated above baseline values (+86.0% and +86.97%, respectively). Sodium selenite (GLY&Na_2_SeO_3_) produced a more pronounced corrective effect, lowering Bax by 21.45% and caspase-3 by 42.83% relative to GLY, bringing these indices closer to control levels (+95.3% and +13.18%, respectively).

Importantly, the QUR-SeNP-treated group (GLY&QUR-SeNPs) exhibited the most substantial modulation of apoptotic dysregulation. Bax expression declined by 43.2% relative to GLY, with only a 41.0% deviation from CONT, while caspase-3 activity was suppressed by 42.29% compared with GLY, nearing physiological levels (+14.24% vs. CONT).

### 2.11. Immunohistochemistry

**Figure 11:**
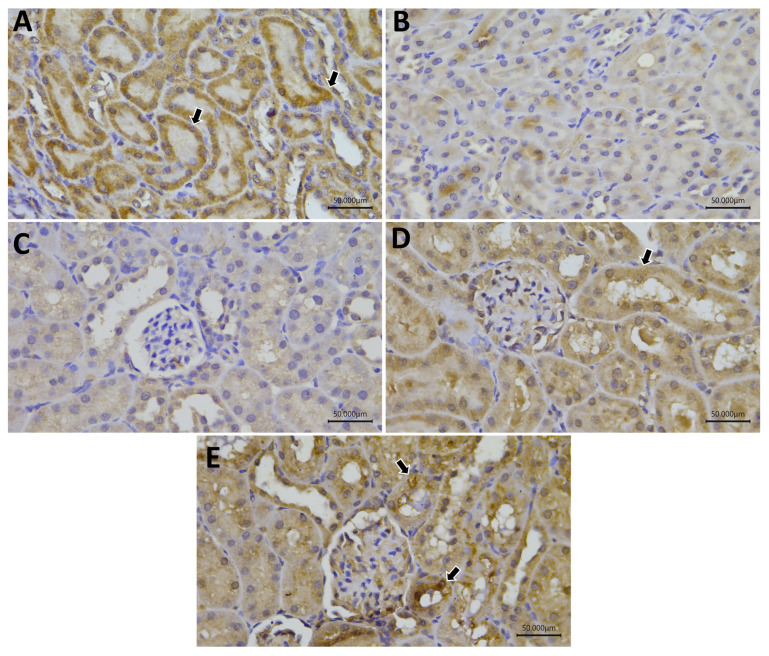
Protective effect of QUR on renal immunoreactivity of Nrf-2 in glycerol-evoked AKI in rats. Severe cytoplasmic reactivity in (**A**) control, (**D**) GLY-Na_2_SeO, and (**E**) GLY-QUR-SeNP groups, while (**B**) GLY and (**C**) GLY-QUR revealed weak Nrf-2 expression. The immunoreactivity of Nrf-2 (arrow) was visualized in the tissues as a brown color developed by DAB chromogen (DAB, ×400).

**Figure 12:**
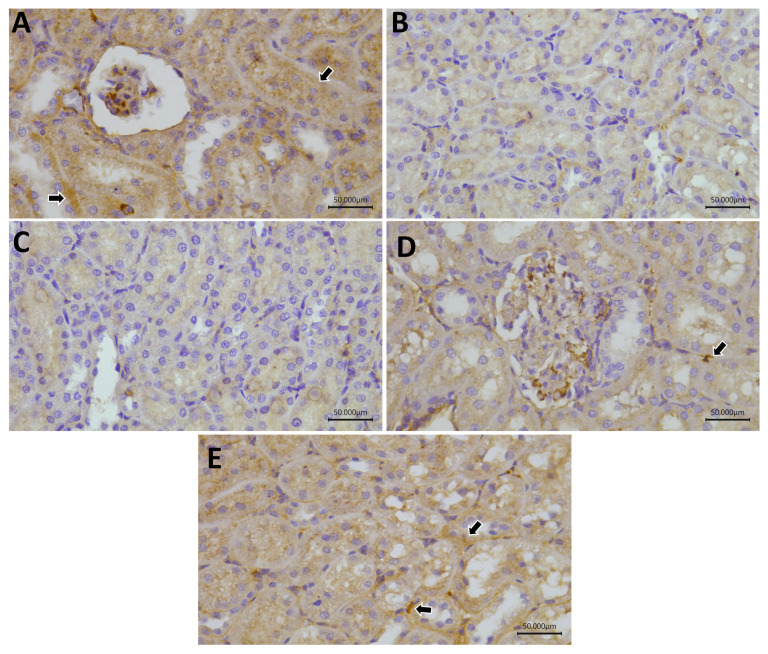
Protective effect of QUR on renal immunoreactivity of FOXP3 in glycerol-evoked AKI in rats. Severe cytoplasmic reactivity in (**A**) control and (**E**) GLY-QUR-SeNPs groups, while (**D**) GLY-Na_2_SeO group revealed moderate expression. (**B**) GLY and (**C**) GLY-QUR revealed weak FOXP3 expression. The immunoreactivity of FoxP3 (arrow) was visualized in the tissues as a brown color developed by DAB chromogen (DAB, ×400).

**Figure 13:**
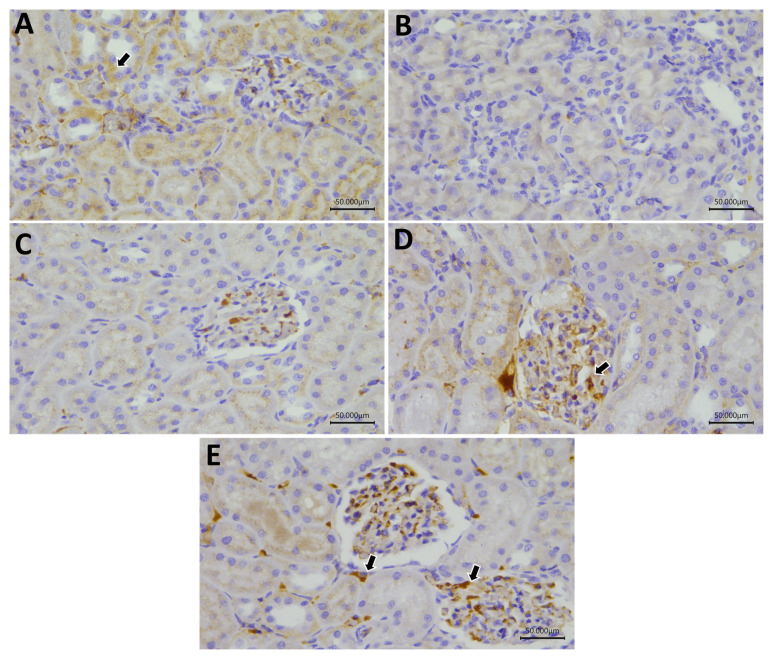
Protective effect of QUR on renal immunoreactivity of Bcl-2 in glycerol-evoked AKI in rats. Severe cytoplasmic reactivity in (**A**) control and (**E**) GLY-QUR-SeNPs groups, while (**D**) Gly-Na_2_SeO group revealed moderate expression. (**B**) GLY and (**C**) GLY-QUR _3_ revealed weak Bcl-2 expression. The immunoreactivity of Bcl-2 (arrow) was visualized in the tissues as a brown color developed by DAB chromogen (DAB, ×400).

**Figure 14:**
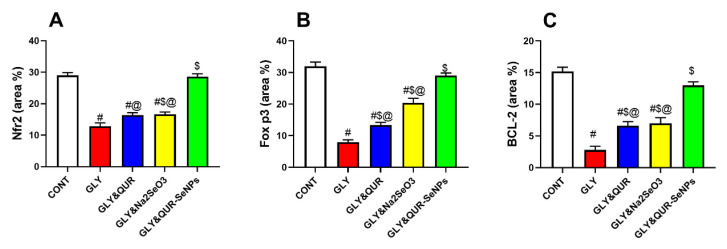
Effect of QUR and QUR-SeNPs administration on (**A**) Nrf-2, (**B**) FoxP3, and (**C**) Bcl-2 in glycerol-evoked AKI in rats. Data are expressed as mean ± SEM (*n* = 8 per group). Statistical annotations: # vs. CONT; $ vs. GLY; @ vs. GLY&QUR-SeNPs (*p* < 0.05).

Regarding Nrf-2, the control, Gly-QUR, and GLY-QUR-SeNP groups revealed marked reactivity, while GLY and GLY-Na_2_SeO_3_ groups revealed weak Nrf-2 expression. Concerning FoxP3 and Bcl-2, the control and GLY-QUR-SeNP groups revealed marked reactivity, while Gly-QUR group showed moderate expression. However, GLY and GLY-Na_2_SeO_3_ groups revealed weak Nrf-2, FoxP3, and Bcl-2 expression. There was remarkable variance between the control and both GLY and GLY-Na_2_SeO_3_ (*p* < 0.01). Interestingly, administration of QUR-SeNPs exhibited marked elevation in Nrf-2, FoxP3, and Bcl-2 reactivity compared to the GLY group (*p* < 0.01) (Figure 11, Figure 12, Figure 13 and Figure 14).

### 2.12. Histopathological Analysis

#### 2.12.1. Skeletal Muscle

**Figure 15:**
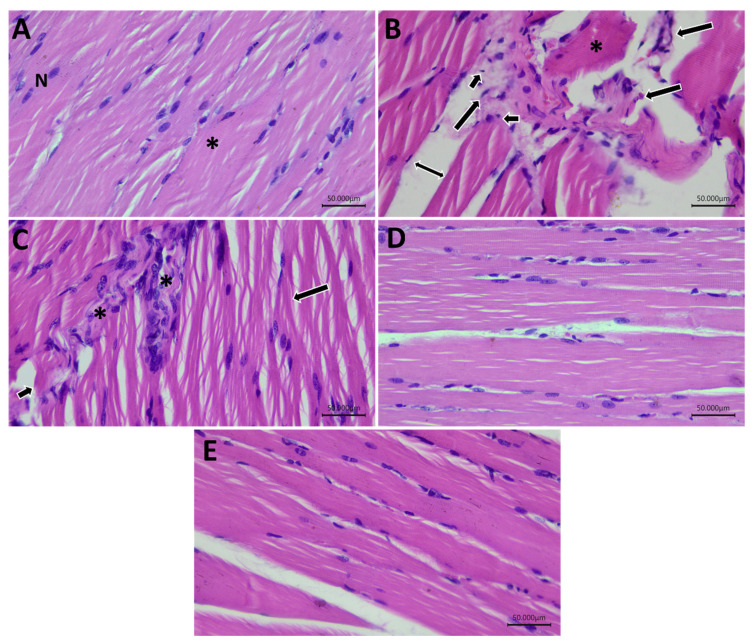
Photomicrograph of gastrocnemius skeletal muscle of all groups. (**A**) Control group showing normal rhabdomyocytes with peripheral nuclei (N) and striated muscle fibers (*). (**B**) GLY group revealed patterns of rhabdomyolysis with focal necrosis with hyaline muscle fiber (*) and necrotic myocytes (long arrow) with pyknotic nuclei (short arrow) infiltrated by mononuclear cells and endomysium edema (double-ended arrow). (**C**) GLY&QUR group exhibited degenerated myocytes (long arrow) with acidophilic cytoplasm (short arrow) with inflammatory infiltration (*). Both (**D**) GLY&Na_2_SeO_3_ and (**E**) GLY-QUR-SeNPs groups showed approximately normal histological appearance (H&E, ×400).

The histopathological assessment of the skeletal muscle of the control group showed normal arrangement of the myocytes with eosinophilic myoplasm filled with myofibrils, peripheral nuclei, and separated with delicate endomysium. The GLY group revealed focal necrosis with rhabdomyolysis and inflammatory mononuclear infiltration and endomysial edema. The GLY&Na_2_SeO_3_ group showed degenerated myocytes with reduced diameters and inflammatory cellular infiltration in the perimysium. Both the GLY&Na_2_SeO_3_ and GLY-QUR-SeNPs groups demonstrated approximately normal skeletal muscle fibers acidophic myoplasm and peripheral nuclei beside the sarcolemma with intercellular endomysial connective tissue and normal perimysium between the fascicles (Figure 15).

#### 2.12.2. Renal Tissue

**Figure 16:**
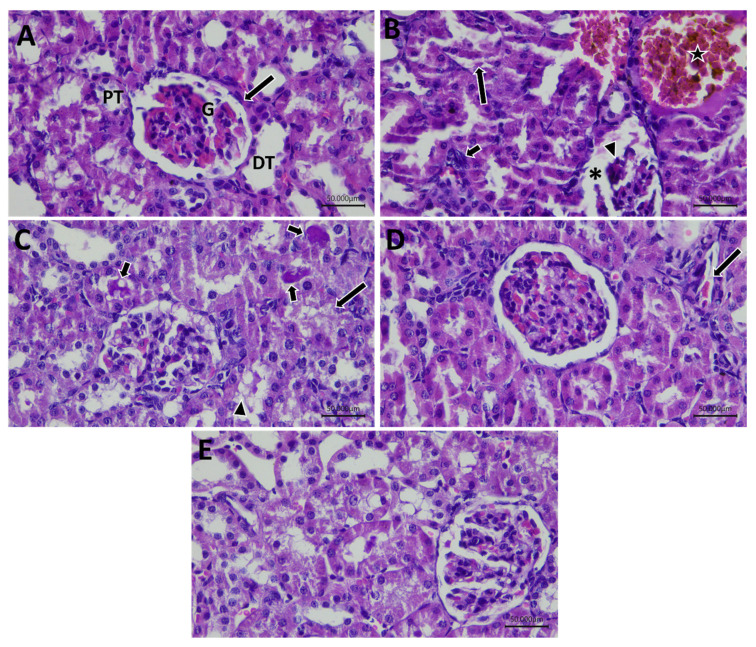
Photomicrograph of renal tissues of all groups. (**A**) Control group showing normal renal cortex with renal corpuscle, glomeruli (G) and glomerular capsule (black arrow), and proximal tubule (PT), and distal tubule (DT). (**B**) GLY group revealed degenerated glomeruli with wide capsular space (*) and necrotic visceral cellular layer (arrow head). Interstitial hemorrhage (star) and vacuolar degeneration of renal tubule with shed cells into the lumen (long arrow) with inflammatory infiltration (short arrow) in the intercapsular space. (**C**) GLY&QUR group exhibited degenerated tubule forming cell (long arrow) with vacuolar degenerated cells (arrow head) and hyaline necrotic material (short arrow). (**D**) GLY&Na_2_SeO_3_ group showed improved cortical appearance with minimal congested capillaries (arrow). (**E**) GLY-QUR-SeNPs group showed approximately normal histological appearance (H&E, ×400).

The control group showed normal renal cortex with normal renal tubules. The GLY group revealed features of renal injury evidenced by focal intratubular hemorrhage, degenerated glomeruli necrotic visceral cellular layer and wide capsular space, and shedding of epithelial cells in the lumen of tubules, in addition, vacuolar degeneration of renal tubule and inflammatory infiltration in the intercapsular space were detected. The GLY&Na_2_SeO_3_ group exhibited a degenerated tubule forming cell with vacuolar degenerated cells and coagulative necrosis was detected. The GLY&QUR group showed improved cortical appearance with congested capillaries. The GLY-QUR-SeNPs group showed approximately normal histological appearance (Figure 16).

## 3. Discussion

Quercetin, a prominent flavonoid with diverse biological activities, exhibits varying predicted binding affinities across different protein targets, as elucidated through molecular docking analyses [18,19]. This computational approach is crucial for understanding protein–ligand interactions and contributes substantially to drug development by revealing prospective drug candidates and their mechanisms of action [20,21,22]. Molecular docking studies have indicated that quercetin demonstrates the most favorable binding interaction with the DQC ubiquitin ligase, evidenced by a Glide Docking score of −6.34 kcal mol^−1^ and an Emodel value of −49.32 kcal mol^−1^ [23]. This suggests a strong predicted affinity for the DQC catalytic site. Ubiquitin ligases, such as E3 ligases, are integral to the ubiquitination process, which marks proteins for degradation by the proteasome [24]. Proteolysis-targeting chimeras (PROTACs), for instance, leverage E3 ubiquitin ligases to induce targeted protein degradation, offering a novel therapeutic strategy, particularly in oncology [23]. The strong binding affinity of quercetin to DQC ubiquitin ligase implies a potential role for quercetin in modulating ubiquitination pathways, which could have implications for cancer therapy or other conditions where protein degradation is critical.

A slightly weaker, yet still notable, binding affinity was predicted for BCL-2 isoform 1, with a Docking score of −5.41 kcal mol^−1^ and an Emodel of −39.13 kcal mol^−1^. BCL-2 proteins are key regulators of apoptosis, and their overexpression is often associated with resistance to chemotherapy in various cancers. For BCL-2, quercetin exhibits a moderate binding affinity with a Docking score of −5.41 kcal/mol [25]. For instance, quercetin’s interaction with BCL-2, PTPN5, and DQC highlights specific binding mechanisms that contribute to its therapeutic potential [25,26]. This interaction is primarily driven by a classic π-stacking interaction with Tyr-28 and three distinct polar contacts within its BH3-mimetic groove. π-stacking interactions, occurring between parallel aromatic rings, are well-established non-covalent forces that stabilize molecular assemblies [27,28]. Tyrosine residues, with their aromatic side-chains, are frequently involved in such interactions [29,30]. The presence of Tyr-28 in the BCL-2 binding groove, forming a π-stacking interaction with quercetin, underscores the importance of aromatic contacts in this specific complex. Polar contacts further augment the binding, contributing to the specificity of quercetin’s interaction with BCL-2. The direct binding of quercetin to Bcl-2 family proteins is known to trigger its pro-apoptotic activity, highlighting the biological relevance of these interactions [25]. Targeting BCL-2 has been a significant strategy in cancer drug development. For example, luteolin and α-linolenic acid have shown strong binding affinities to AKT1, which is involved in cell survival pathways that can interact with BCL-2 family proteins [31]. Specifically, when quercetin binds to BCL-2, it forms 3D contacts with residues. The binding interactions include a π–π-stack with Tyr-28, hydrogen bonds with Asp-31, Gly-27, and Asn-39, and Van der Waals interactions with His-20. This moderate affinity, characterized by a classic π-stack and three polar contacts within the BH3-mimetic groove, contributes to its pro-apoptotic activity [25]. The Docking score for quercetin with BCL-2 (1G5M) is −5.41 kcal/mol, and the Glide Emodel score is −39.13 kcal/mol. The direct binding of quercetin to BCL-2 family proteins can trigger pro-apoptotic activity, which is particularly relevant given that anti-apoptotic BCL-2 members are often overexpressed in various cancers, making them attractive therapeutic targets [32]. While quercetin’s binding to BCL-2 is less potent than to DQC, it still indicates a possible influence on apoptotic pathways. In contrast, the interaction with human PTPN5 yielded a Docking score of −5.35 kcal mol^−1^, comparable to BCL-2, but was accompanied by a more negative Emodel of −53.40 kcal mol^−1^. Protein tyrosine phosphatases (PTPs), including PTPN5, regulate cellular signaling by dephosphorylating tyrosine residues on proteins. Dysregulation of PTPs is implicated in various diseases, including neurological disorders and cancer. For instance, the molecular docking results for various compounds with different target proteins, including ETS1, shows Fluzoparib with a Docking score of −9.2 kcal/mol, indicating a strong binding interaction [33]. The Emodel value is a composite scoring function that considers binding energy and conformational strain, providing a more comprehensive measure of binding stability [21]. For PTPN5, quercetin establishes contacts with His-312, Arg-314, Asp-510, Tyr-334, Lys-343, and Glu-342. The binding involves hydrogen bond acceptors with Arg-314 and His-312, a bifurcated hydrogen bond donor with Glu-342 and Asp-510, a π-stack with Tyr-334, and electrostatic interactions with Lys-343. The Docking score for quercetin with PTPN5 (2BIJ) is −5.35 kcal/mol, and the Glide Emodel score is −53.40 kcal/mol. A more negative Emodel for PTPN5 suggests a particularly stable interaction, despite a Docking score similar to BCL-2. These results collectively propose a modest in silico selectivity of quercetin for the DQC ubiquitin ligase binding site over PTPN5 and BCL-2 proteins. Molecular docking, while powerful, is a computational prediction method [34,35]. The interaction of quercetin with DQC demonstrates the highest affinity among the three targets mentioned. Key residues involved include His-59, Glu-658, Cys-653, Asp-654, Met-651, Leu-650, and Arg-652. The strong binding is attributed to a dense network of four direct hydrogen bonds, along with robust π/alkyl contacts. Specific interactions include chelation of structural water by Asp-654, a hydrogen bond donor interaction with His-59, a salt-bridge-like interaction with Arg-652, a hydrogen bond acceptor interaction with Met-651, and Van der Waals interactions with Leu-650, Val-656, and Met-651. The Docking score for quercetin with DQC (6WCQ) is −6.34 kcal/mol and the Glide Emodel score is −49.32 kcal/mol. The superior affinity for DQC is structurally rationalized by an extensive hydrogen bonding network and significant π-stacking/Van der Waals interactions.

The accuracy of binding affinity predictions has been enhanced by recent advances in 3D structure-based deep learning approaches, which complement physics-based computational modeling like molecular docking. Nevertheless, comprehensive validation typically requires experimental assays, such as surface plasmon resonance or isothermal titration calorimetry, to confirm binding and assess functional implications. For example, kinetic analysis of MYH9 and quercetin interaction revealed a dissociation constant (Kd) of 90.1 ± 9.68 μM [36].

This study was designed to explore the nephroprotective potential of selenium nanoparticles biosynthesized using quercetin (QUR-SeNPs) against experimentally rhabdomyolysis-induced acute kidney injury (AKI). The results demonstrated that QUR-SeNPs effectively ameliorated renal functional, biochemical, and molecular disturbances through their synergistic antioxidant, anti-inflammatory, and anti-apoptotic properties, surpassing the effects of either quercetin or selenium alone.

Renal injury was evident in the intoxicated group, as reflected by the marked elevation of serum urea and creatinine together with significant increases in kidney injury biomarkers KIM-1 and NGAL, this is consistent with Khames et al. [37], Jana et al. [38], and Umar Ijaz et al. [39]. These findings confirm severe tubular damage and loss of glomerular filtration integrity. KIM-1 and NGAL are highly sensitive markers of proximal tubular injury and early nephrotoxicity, and their upregulation parallels the degree of tubular epithelial damage reported in previous study [37]. Treatment with QUR-SeNPs restored renal function and markedly decreased serum urea and creatinine, renal KIM-1, and NGAL levels. These results indicate effective suppression of tubular injury and ferroptotic cell death, in agreement with studies showing that selenium and flavonoid nanoformulations reinforce antioxidant selenoprotein expression and mitigate renal oxidative injury. The superior efficacy of QUR-SeNPs can be attributed to their unique nano-architecture, which enhances cellular uptake, prolongs systemic circulation, and facilitates controlled intracellular release of both selenium and quercetin. Upon internalization, selenium acts as a cofactor for GPX4 biosynthesis, directly strengthening the enzymatic detoxification of lipid hydroperoxides, whereas quercetin scavenges reactive oxygen and nitrogen species and activates Nrf2-dependent antioxidant pathways [20,38]. The combined effect results in restoration of redox homeostasis, stabilization of cellular membranes, and prevention of ferroptosis-driven tubular damage. Moreover, the nano-formulation shields quercetin from rapid metabolism, ensuring sustained bioactivity and synergistic reinforcement of selenoprotein-mediated cytoprotection within renal tissue. Acute kidney injury (AKI) was accompanied by significant oxidative derangements, as evidenced by increased MDA and NO levels and decreased antioxidant defenses (GSH, SOD, CAT, and GPx activities). In parallel, the reduction in FOXP3 expression primarily reflects impaired regulatory T-cell function rather than a defect in antioxidant capacity. FOXP3 is a key transcription factor that maintains the suppressive activity of Tregs, which play a central role in controlling excessive immune activation and preventing tissue-damaging inflammation. Thus, its downregulation in AKI suggests a loss of immunological restraint, allowing pro-inflammatory pathways to become further amplified within renal tissue. This diminished Treg signaling can exacerbate cytokine production, promote leukocyte infiltration, and intensify tubular injury. Therefore, the observed decrease in FOXP3 is best interpreted as an indicator of immune dysregulation contributing to renal inflammation, rather than a direct marker of oxidative imbalance. The depletion of GPX4—a selenoenzyme central to lipid peroxide detoxification suggests enhanced ferroptotic injury, consistent with literature demonstrating GPX4 downregulation as a hallmark of oxidative nephropathy [39]. Previous studies have confirmed that OS constitutes a central mechanism in nephrotoxin-induced renal injury, resulting in mitochondrial dysfunction and cellular necrosis [40,41,42]. Administration of QUR-SeNPs significantly restored antioxidant enzyme activities and reduced lipid peroxidation. The observed overcompensation in antioxidant levels in the QUR-SeNP-treated group supports a strong redox-modulatory effect of the nanocomposite. The enhanced efficacy compared with native quercetin may be attributed to the improved stability and bioavailability of the nanoparticle formulation, which facilitates sustained ROS scavenging and upregulation of Nrf2-mediated antioxidant pathways.

The intoxicated group exhibited pronounced renal inflammation characterized by significant upregulation of TNF-α, IL-1β, and IL-6, confirming the activation of pro-inflammatory cascades. Such cytokine elevations are mediated through NF-κB activation and are known to propagate renal fibrosis and endothelial dysfunction [43,44]. The current findings are consistent with previous reports describing cytokine-driven renal injury following oxidative insult [45,46,47]. QUR-SeNP treatment markedly reduced all inflammatory mediators, indicating potent anti-inflammatory action. This suppression is likely attributed to quercetin’s inhibitory effects on NF-κB and iNOS expression, potentiated by selenium’s role in modulating immune redox signaling, Together, these effects interrupt the NF-κB–cytokine feedback loop, thereby preventing the amplification of renal inflammation. Notably, QUR-SeNPs achieved greater suppression of cytokines than either QUR or Se alone, reflecting synergistic crosstalk between their antioxidant and anti-inflammatory mechanisms, which collectively hindered further progression of nephroinflammation. AKI induced a significant increase in pro-apoptotic proteins Bax and caspase-3, with concomitant downregulation of the anti-apoptotic marker Bcl-2, confirming activation of the intrinsic mitochondrial apoptotic pathway. Consistent with the expected apoptotic response in AKI, glycerol administration caused a significant elevation in both Bax expression and caspase-3 activity, indicating activation of the intrinsic mitochondrial apoptotic pathway. As shown in Figure 10, QUR-SeNPs markedly reduced Bax and caspase-3 levels compared with the GLY group, demonstrating strong anti-apoptotic activity. Notably, the reduction produced by QUR-SeNPs was significantly greater than that observed with quercetin or selenium alone, reflecting a synergistic protective effect of the nanocomposite on mitochondrial integrity and apoptosis suppression. This pattern mirrors prior studies showing OS-driven apoptosis as a principal mechanism of renal parenchymal loss, and this is in line with Ren et al. [48], Ortega [49], and Sindhughosa and Pranamartha [50]. QUR-SeNP administration restored cellular homeostasis by reducing Bax and caspase-3 while elevating Bcl-2 expression, suggesting preserved mitochondrial integrity and inhibition of apoptotic signaling. The superior anti-apoptotic efficacy of QUR-SeNPs compared with the parent compounds likely stems from improved cellular penetration and controlled release, ensuring continuous mitigation of oxidative and inflammatory triggers of apoptosis. These findings align with studies reporting that selenium nanoparticles and quercetin derivatives synergistically suppress renal apoptosis by stabilizing mitochondrial membranes and maintaining ATP synthesis [51,52].

**Figure 17:**
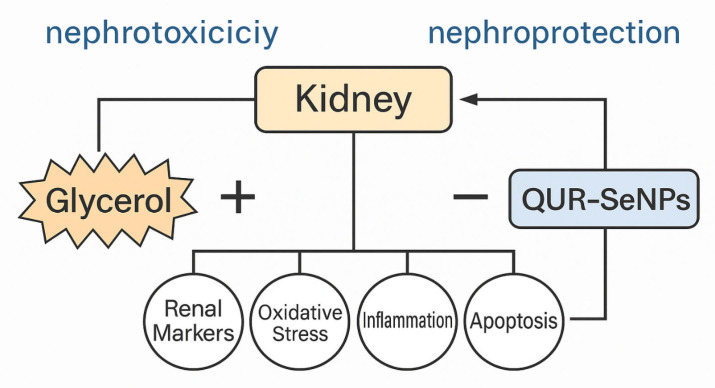
The proposed mechanism of QUR-SeNPs nephroprotective effect.

Collectively, the results delineate a clear mechanistic interplay among OS, inflammation, and apoptosis in the pathogenesis of acute kidney injury (AKI). Excessive ROS production triggered lipid peroxidation and depletion of GPX4, which in turn activated NF-κB signaling and cytokine overexpression, establishing a vicious cycle that culminated in mitochondrial dysfunction and apoptotic cell loss. The concurrent elevation of KIM-1 and NGAL further substantiates tubular epithelial damage secondary to these cascades. In parallel, the observed suppression of FOXP3 suggests impaired transcriptional regulation of antioxidant and survival genes, thereby amplifying oxidative and apoptotic signaling. QUR-SeNPs effectively disrupted this pathological triad by reinforcing antioxidant defenses (via GPX4, FOXP3, and Nrf2 activation), suppressing inflammatory mediators, and preserving anti-apoptotic signaling (Figure 17). The convergence of these protective effects underscores the therapeutic potential of QUR-SeNPs as multifunctional nephroprotective agents, a conclusion consistent with previous reports on FOXP3-dependent redox homeostasis in renal protection. The present study utilized an acute toxicity model in rats, which may not fully emulate chronic or multifactorial renal injury in humans. Future studies incorporating chronic exposure models and ultrastructural imaging are warranted to validate the translational potential of QUR-SeNPs.

## 4. Materials and Methods

### 4.1. Molecular Docking

Molecular docking simulations were meticulously performed using the Schrödinger Glide software suite (V. 2025.2), employing its standard-precision (SP) mode [53,54]. The three-dimensional crystal structures of human PTPN5 (PDB 2BIJ), DQC ubiquitin ligase (PDB 6WCQ) [55], and BCL-2 isoform 1 (PDB 1G5M) were retrieved from the Protein Data Bank and utilized directly without further structural refinement [56]. The ligand, quercetin, was subjected to preparation using the LigPrep workflow within Schrödinger, which involved generating possible tautomeric and ionization states at a target pH of 7.0 ± 2.0, and optimizing the 3D geometry. For each protein, a single receptor grid was precisely generated. In the case of PTPN5 and BCL-2, the grids were centered on the co-crystallized ligand binding sites to ensure accurate localization of the docking space. For the DQC ubiquitin ligase, the grid was specifically positioned over the known catalytic site. A single pose per target was ultimately retained for subsequent analysis. The primary metrics recorded for each docking event were the Glide gscore (Docking score in kcal mol^−1^), representing the overall binding affinity, and the Emodel value (kcal mol^−1^), which provides an estimate of the ligand–protein interaction energy, offering a complementary perspective on binding stability [56].

### 4.2. Synthesis of Selenium Nanoparticles

Quercetin-mediated selenium nanoparticles (QUR-SeNPs) were prepared via a green reduction approach in which quercetin served simultaneously as the reducing agent. The synthesis was performed under aseptic conditions within a sterile laminar flow cabinet. Briefly, 100 mL of an aqueous sodium selenite solution (5.78 mM) was prepared and added dropwise to an equal volume of quercetin dissolved in methanol (16.5 mM), which had been sterilized through a 0.20 μm syringe filter. The reaction mixture was maintained at 60–70 °C under continuous magnetic stirring and protected from light to prevent photodegradation. A progressive color transition from yellow to dark brown over approximately 5 h signified the successful reduction of selenium ions and formation of QUR-SeNPs [57].

#### Characterization of Selenium Nanoparticles

The physicochemical characteristics of the biosynthesized SeNPs were evaluated using a combination of dynamic light scattering (DLS), zeta potential analysis, and high-resolution Transmission Electron Microscopy (TEM). Particle size distribution and surface charge were measured using a Zetasizer Nano ZS90 (Malvern Panalytical Ltd., Malvern, UK) at the Faculty of Engineering, Helwan University (Cairo, Egypt). Prior to analysis, nanoparticle suspensions were diluted 1:10 in deionized water and sonicated for 5 min to minimize transient aggregation. DLS measurements were conducted at 25 °C in disposable polystyrene cuvettes, providing the Z-average hydrodynamic diameter and polydispersity index (PDI). Zeta potential values were obtained by electrophoretic light scattering using folded capillary zeta cells, with all measurements performed in triplicate to ensure reproducibility. These analyses confirmed the colloidal stability of the SeNPs.

Morphological properties, particle architecture, and size distribution were further examined using high-resolution TEM (JEOL JEM-2100, JEOL Ltd., Tokyo, Japan) at Al-Azhar University (Cairo, Egypt). For imaging, a drop of the nanoparticle suspension was placed on a carbon-coated 300-mesh copper grid, it was allowed to adsorb for 2 min, and excess liquid was removed gently with filter paper. The grid was subsequently air-dried under a vacuum. TEM imaging was conducted at an accelerating voltage of 200 kV, allowing visualization of ultrastructural details. The obtained micrographs revealed the nanoscale dimensions, uniform distribution, spherical morphology, and surface integrity of the SeNPs, confirming successful synthesis and quercetin-mediated stabilization.

FTIR spectroscopy was used to identify the distinctive functional groups of pure QUR and explore its interaction with SeNPs in the QUR-SeNPs complex. The spectra were obtained from 4000 to 400 cm^−1^. For the analysis, samples were prepared using the traditional KBr pellet procedure, which involves carefully mixing a tiny amount of the sample with dry potassium bromide (KBr) and compressing it into a thin, clear pellet. To achieve a good signal-to-noise ratio, the instrument was tuned to a resolution of 4 cm^−1^ with an average of 32 scans each spectrum. The obtained spectra were plotted as transmittance (%) vs. wave number (cm^−1^). The peak positions were found and attributed to their respective chemical vibrations [58].

### 4.3. Experimental Animals and Study Design

The present investigation was undertaken to assess the nephroprotective potential of quercetin-mediated, green-synthesized selenium nanoparticles (QUR-SeNPs) against glycerol-induced rhabdomyolysis leading to acute kidney injury (AKI). Forty healthy adult male albino rats, weighing 100–120 g, were employed in the experiment. Animals were randomly distributed into five groups, each comprising eight rats. They were maintained under standardized laboratory conditions, including a temperature of 22 ± 2 °C, appropriate humidity levels, and a controlled 12 h light/12 h dark photoperiod. Standard laboratory chow and water were provided, but water was deprived for 24 h before glycerol injection. The animal study protocol was approved by the Sinai University Research Ethics Committee (SU. REC.2025. 76 A) (1 October 2025). Animal pain or suffering was minimized as much as possible during experimentation. All processes of animal experimentation were executed as stated in the ARRIVE guidelines, in accordance with the UK Animals Act, 1986, and approved by the Research Ethics Committee. We ensure that all aspects of the research comply with the guidelines of the ethical review committee and international ethical research standards.

The grouping of animals was structured as follows:

Control group (CONT, no = 8): Animals of this group were orally administrated with normal saline solution (0.9% NaCl) for 15 consecutive days, at day 14 animals of this group were injected with intramuscular saline single dose (10 mg/kg). Glycerol group (GLY, no = 8): Animals of this group were orally administrated with normal saline (0.9% NaCl) for 15 consecutive days, at day 14, animals of this group will receive single dose of intramuscular injection of 50% glycerol (10 mg/kg/day) [59,60]. Quercetin group (GLY&QUR, no = 8): Animals of this group were orally administrated with quercetin daily for 15 days (50 mg/kg/day) [61], at day 14, animals of this group will receive single dose of im. injection of 50% glycerol (10 mg/kg/day) [62]. Sodium selenite group (GLY&Na_2_SeO_3_, no = 8): Animals of this group were orally administrated with sodium selenite daily for 15 days (0.5 mg/kg/day) [63], at day 14, animals of this group will receive single dose of im. injection of 50% glycerol (10 mg/kg/day) [59]. Selenium nano-particles biosynthesized using quercetin group (GLY&QUR-SeNPs, no = 8): Animals of this group were orally administrated with selenium nanoparticles synthesized using quercetin daily for 15 days (0.5 mg/kg/day) [63], at day 14, animals of this group will receive single dose of im. injection of 50% glycerol (10 mg/kg/day) [59].

### 4.4. Kidney Weight Estimation

Relative kidney weight was calculated using the following formula, which expresses the organ weight in proportion to the body weight of the animal [64]:

$$Relative\;kidney\;weight=\frac{Left\;kidney\;wt\;(g)}{Body\;wieght\;(g)}\times 100$$

### 4.5. Tissue Collection and Sample Preparation

The rats received an intraperitoneal injection (1 mL) of anesthesia (0.3 mL of xylazine and 0.7 mL of ketamine) and were sacrificed using a sterile surgical blade. Following decapitation, blood samples were allowed to clot at room temperature for 15 min and subsequently centrifuged at 3000 rpm for 10 min at 4 °C to obtain a serum, which was utilized for evaluating renal function indices, creatine kinase, and lactate dehydrogenase activities. Kidney tissues were processed separately, where 10% (*w*/*v*) homogenates were prepared in phosphate-buffered saline (PBS; 10 mM phosphate buffer containing 138 mM NaCl and 2.7 mM KCl, pH 7.4). Homogenates were centrifuged at 10,000 rpm for 10 min at 4 °C, and the resulting supernatants were employed for the determination of OS biomarkers, inflammatory mediators, and related biochemical parameters. All assays were conducted using commercially available ELISA and colorimetric kits, strictly following the manufacturers’ instructions. For histological and immunohistochemical studies, selected kidney samples were fixed in 10% neutral buffered formalin, embedded in paraffin, sectioned, and processed for standard staining and microscopic examination.

### 4.6. Assessment of Kidney Function Test Level

Renal function was assessed by quantifying serum creatinine and urea were determined with Biodiagnostic assay kits (Giza, Egypt), following the classical procedure [65]. The principle is based on the measured spectrophotometrically at 505 nm. Activities were calculated using standard calibration curves.

### 4.7. Creatine Kinase (CK) and Lactate Dehydrogenase (LDH) Assay

Serum activities of creatine kinase (CK) and lactate dehydrogenase (LDH) were determined using commercially available colorimetric diagnostic kits (Biodiagnostic, Giza, Egypt) according to the manufacturer’s protocols. CK activity was assessed by the kinetic UV method, which measures the rate of NADPH oxidation during the conversion of creatine phosphate to creatine in the presence of ADP. The decline in absorbance was monitored spectrophotometrically at 340 nm, and enzyme activity was expressed in U/L [66]. LDH activity was determined by the lactate-to-pyruvate conversion method, where the oxidation of NADH to NAD^+^ was measured kinetically at 340 nm. Both assays were conducted in duplicate under standardized laboratory conditions to ensure reproducibility and accuracy. Results were expressed as mean values of enzyme activities, providing sensitive biochemical indices for evaluating tissue injury and renal dysfunction associated with glycerol-induced nephrotoxicity through inducing rhabdomyolysis leading to acute kidney injury (AKI) and for assessing the potential protective efficacy of the tested treatments [67].

### 4.8. Assessment of Oxidant/Antioxidant Biomarkers

Renal oxidative status was evaluated in tissue homogenates by quantifying malondialdehyde (MDA), nitric oxide (NO), reduced glutathione (GSH), superoxide dismutase (SOD), and catalase (CAT). Lipid peroxidation was assessed by measuring MDA, the terminal product of peroxidative degradation of lipids, using the thiobarbituric acid reactive substances (TBARS) method [68], with absorbance recorded at 535 nm. Nitric oxide was determined via the Griess reaction, estimating total nitrite/nitrate concentrations at 540 nm as previously described [69]. Reduced glutathione levels were measured following the method of [70], employing 5,5′-dithiobis-(2-nitrobenzoic acid) (DTNB) and recording the absorbance of the generated chromophore at 412 nm. Antioxidant enzyme activities were assayed spectrophotometrically: SOD activity was determined by its inhibitory effect on nitroblue tetrazolium reduction [71] at 560 nm, while CAT activity was assessed via tracking the rate of hydrogen peroxide breakdown at 240 nm, according to Ciralone [72].

### 4.9. Inflammatory/Anti-Inflammatory Marker Evaluation

The levels of TNF-α, IL-6, and NF-κB were quantified using highly sensitive sandwich ELISA kits (Abcam, Cambridge, UK), following the manufacturers’ instructions. Briefly, capture antibodies pre-coated onto microplates bound the respective analytes, which were subsequently detected with biotinylated secondary antibodies and horseradish peroxidase (HRP)-conjugated streptavidin [73,74]. The enzymatic reaction was visualized using tetramethylbenzidine (TMB) substrate, it was terminated with an acidic stop solution, and absorbance was recorded at 450 nm. Concentrations were determined from standard calibration curves generated by serial dilutions of recombinant rat cytokines [75]. The assay kits employed included ab213899 for TNF-α, ab10076 for IL-6, and MBS2505513 for NF-κB, all of which have been validated for reliable assessment of inflammatory mediators in rodent models [76].

### 4.10. Gene Expression Analysis

Quantitative reverse transcription polymerase chain reaction (RT-qPCR) was employed to assess gene expression. Total RNA was isolated from renal tissues and subsequently reverse-transcribed into complementary DNA. The transcriptional profile of nuclear factor kappa B (*NF-κB*) and *cystatin-C* was determined using specific primer sets, with *GAPDH* utilized as the endogenous control for data normalization. Relative expression levels were calculated according to the 2^−ΔΔCt^ method [77,78], as presented in Table 3.

### 4.11. Immunohistochemical Analysis of Nrf2, Bcl-2, and FOXP3

Immunohistochemistry was performed on paraffin-embedded renal sections (4–5 µm) to assess the expression of Nrf2, Bcl-2, and FOXP3. As part of these procedures, tissue sections were dewaxed and rehydrated, and antigens were removed from them by boiling them in citrate buffer (pH 6.0). Hydrogen peroxide (3%) was applied for 20 min to quench tissue endogenous peroxidase activity. Then, 5% bovine serum albumin was used as a blocking agent in a humidified chamber to avoid the non-specific binding of antibodies to tissue proteins. Next, 4 µm sections of testicular tissues were immunostained with anti-Nrf2, anti-Bcl-2, and anti-Foxp3 primary antibodies for 90 min. Followed by exposure to species with matched biotinylated secondary antibodies and streptavidin peroxidase conjugates. Immunoreactivity was visualized using 3,3′-diaminobenzidine (DAB) as the chromogenic substrate, and nuclei were counterstained with hematoxylin. Stained sections were examined under light microscopy to evaluate localization and intensity of protein expression [79,80,81].

### 4.12. Quantitative Assessment of IHC Staining

This step is an effective tool for studying protein location inside tissue. Semi-quantitative IHC is carried out using ImageJ Fiji software version 1.2 (without the use of any special plugin). Deconvolution and downstream analysis were completed. Nrf-2, BCL-2, and FOXP3 immunoreaction area % was measured at magnification ×400 for all groups [82,83].

### 4.13. Histopathological Examination

Kidney tissue and skeletal muscle specimens were fixed, processed, and stained with hematoxylin and eosin (H&E) for the assessment of general histoarchitecture, including nephrocytes arrangement, vascular integrity, and evidence of degenerative or necrotic changes [84,85].

### 4.14. Statistical Analysis

Data is estimated as mean ± SEM. Statistical comparisons across groups were made via one-way ANOVA followed by Tukey’s post hoc test. Analyses were performed using GraphPad Prism software (version 9.0; GraphPad Software, San Diego, CA, USA). *p*-values < 0.05 indicated statistical significance. The percentage change in comparison to the control group is determined employing the following equation:

$$\%\;of\;Difference=\frac{\mathrm{Treatrd}\;\mathrm{value}-\mathrm{control}\;\mathrm{value}}{\mathrm{Control}\;\mathrm{value}}\times 100$$

## 5. Conclusions

Eco-synthesized QUR-SeNPs provided strong nephroprotection by restoring redox balance, suppressing inflammatory signaling, and reducing mitochondrial apoptosis, thereby preserving overall renal integrity. Their superior performance compared with QUR or selenium alone reflects a clear synergistic enhancement in antioxidant capacity, cytokine modulation, and anti-apoptotic activity. By reinforcing endogenous defenses, stabilizing mitochondrial function, and limiting inflammatory damage, QUR-SeNPs demonstrate a robust and coordinated protective effect. Furthermore, QUR-SeNPs mitigated apoptotic injury through downregulation of Bax and caspase-3 and upregulation of the anti-apoptotic protein Bcl-2. These coordinated molecular actions underscore the superior therapeutic potential of QUR-SeNPs compared to quercetin or selenium alone. These findings support QUR-SeNPs as a promising multifunctional nanotherapeutic platform for mitigating acute renal injury.

## Figures and Tables

**Table 1:** The best-scoring pose for each target is summarized below (all energies in kcal mol^−1^ unless noted).

Target (PDB-ID)	Docking Score	Glide Emodel
Kim1-PTPN5 (2BIJ)	−5.35	−53.40
Nrf2-DQC (6WCQ)	−6.34	−49.32
BCL-2 (1G5M)	−5.41	−39.13

**Table 2:** Summary of quercetin’s binding site interactions.

Target (PDB-ID)	3D Contacts (≤3.5 Å)	Binding Interactions	Structural Rationale for Affinity
BCL-2 (1G5M)	Tyr-28, Gly-27, Asp-31, Asn-39, His-20	π–π-stack (Tyr-28); H-bonds (Asp-31, Gly-27, Asn-39); VdW (His-20)	Moderate affinity driven by classic π-stack and three polar contacts in the BH3-mimetic groove.
PTPN5 (2BIJ)	His-312, Arg-314, Asp-510, Tyr-334, Lys-343, Glu-342	H-bond acceptors (Arg-314, His-312); bifurcated H-bond donor (Glu-342, Asp-510); π-stack (Tyr-334); electrostatic (Lys-343)	Slightly weaker affinity, but the pose is anchored by two arginine grips compensating for a smaller hydrophobic surface.
DQC (6WCQ)	His-59, Glu-658, Cys-653, Asp-654, Met-651, Leu-650, Arg-652	Chelation of structural water (Asp-654); H-bond donor (His-59); salt-bridge-like (Arg-652); H-bond acceptor (Met-651); VdW (Leu-650, Val-656, Met-651)	Highest affinity due to a dense network of four direct H-bonds plus robust π/alkyl contacts.

**Table 3:** Primer sequences of genes analyzed by RT-qPCR.

	Forward Sequence	Reverse Sequence	ACC. Number
*Cystatin-C*	CTTGGGCTAGAGAGCGGGA	TGAAGCACGGGTGAGTCTTC	NM_012839.2
*NFκB*	GTCTCAAACCAAACAGCCTCAC	CAGTGTCTTCCTCGACATGGAT	NM_199267.2
*GAPDH*	ATGGTGAAGGTCGGTGTGAACG	TGGTGAAGACGCCAGTAGACTC	NM_017008.4

## Data Availability

The original contributions presented in this study are included in the article. Further inquiries can be directed to the corresponding authors.

## Abbreviations

The following abbreviations are used in this manuscript:

AKI	Acute kidney injury
ICU	Intensive care unit
QUR	Quercetin
GLY	Glycerol
SeNPs	Selenium nanoparticles
im.	Intramuscular
DNA	Deoxyribonucleic acid
ROS	Reactive oxygen species
OS	Oxidative stress
BCL-2	B-cell lymphoma-2
Caspase-3	Cysteine aspartate-specific protease 3
DQC	Dimerization Quality Control
Kim-1	Kidney Injury Molecule-1
PTPN5	Protein tyrosine phosphatase non-receptor type 5
Nrf2	Nuclear factor erythroid 2-related factor 2
Asp	Aspartic acid
Gly	Glycine
Asn	Asparagine
Arg	Arginine
His	Histidine
Glu	Glutamic acid
Try	Tyrosine
Met	Methionine
Leu	Leucine
Val	Valine
PDI	Polydispersity index
DLS	Dynamic light scattering
TEM	Transmission Electron Microscopy
FT-IR	Fourier Transform Infrared
CONT	Control
LDH	Lactate dehydrogenase
CK	Creatine kinase
BUN	Blood urea nitrogen
NOS2	Nitric oxide synthase 2
MDA	Malondialdehyde
LPO	Lipid peroxidation
NO	Nitroxide
GSH	Glutathione synthetase
SOD	Superoxide dismutase
CAT	Catalase
TNF-α	Tumor necrosis factor alpha
NF-κB	Nuclear factor kappa B
IL-1β	Interlukin-1β
Bax	Bcl-2 associated X-protein
GPx	Glutathione peroxidase
PDB	Protein Data Bank
FOXP3	Forkhead box P3
DAB	3,3′-diaminobenzidine
ETS1	ETS proto-oncogene 1
NGAL	Neutrophil gelatinase-associated lipocalin
iNOS	Inducible nitric oxide synthase
ATP	Adenosine triphosphate
NADH	Nicotinamide adenine dinucleotide
TBARS	Thiobarbituric acid reactive substances
HRP	Horseradish peroxidase
ELISA	Enzyme-Linked Immunosorbent Assay
RT-qPCR	Quantitative reverse transcription polymerase chain reaction
GAPDH	Glyceraldehyde-3-phosphate dehydrogenase
IHC	Immunohistochemistry
SEM	Standard Error of the Mean

## References

[1] Connor S., Li T., Qu Y., Roberts R.A., Tong W. (2024). Generation of a Drug-Induced Renal Injury List to Facilitate the Development of New Approach Methodologies for Nephrotoxicity. Drug Discov. Today.

[2] Hosohata K. (2016). Role of Oxidative Stress in Drug-Induced Kidney Injury. Int. J. Mol. Sci..

[3] Leng X.-Y., Liu C.-N., Wang S.-C., Peng H.-D., Wang D.-G., Pan H.-F. (2022). Comparison of the Efficacy of Nonsteroidal Anti-Inflammatory Drugs and Opioids in the Treatment of Acute Renal Colic: A Systematic Review and Meta-Analysis. Front. Pharmacol..

[4] Rong J., Zhang Z., Peng X., Li P., Zhao T., Zhong Y. (2024). Mechanisms of Hepatic and Renal Injury in Lipid Metabolism Disorders in Metabolic Syndrome. Int. J. Biol. Sci..

[5] Ali H.H., Ahmed Z.A., Aziz T.A. (2022). Effect of Telmisartan and Quercetin in 5 Fluorouracil-Induced Renal Toxicity in Rats. J. Inflamm. Res..

[6] Balkrishna A., Gohel V., Pathak N., Joshi M., Singh R., Kumari A., Dev R., Varshney A. (2024). Renogrit Selectively Protects against Cisplatin-Induced Injury in Human Renal Tubular Cells and in Caenorhabditis Elegans by Harmonizing Apoptosis and Mitophagy. Sci. Rep..

[7] Lin X., Jin H., Chai Y., Shou S. (2022). Cellular Senescence and Acute Kidney Injury. Pediatr. Nephrol..

[8] Shi M., Mobet Y., Shen H. (2024). Quercetin Attenuates Acute Kidney Injury Caused by Cisplatin by Inhibiting Ferroptosis and Cuproptosis. Cell Biochem. Biophys..

[9] Xu X., Zeng T., Chen S., Tian N., Zhang C., Chen Y., Deng S., Mao Z., Liao J., Zhang T. (2025). Acute Kidney Injury: Pathogenesis and Therapeutic Interventions. Mol. Biomed..

[10] Burki S., Burki Z.G., Asghar M.A., Ali I., Zafar S. (2021). Phytochemical, Acute Toxicity and Renal Protective Appraisal of Ajuga Parviflora Hydromethanolic Leaf Extract against CCl4 Induced Renal Injury in Rats. BMC Complement. Med. Ther..

[11] Pethő Á.G., Tapolyai M., Csongrádi É., Orosz P. (2024). Management of Chronic Kidney Disease: The Current Novel and Forgotten Therapies. J. Clin. Transl. Endocrinol..

[12] Jin Q., Liu T., Qiao Y., Liu D., Yang L., Mao H., Ma F., Wang Y., Peng L., Zhan Y. (2023). Oxidative Stress and Inflammation in Diabetic Nephropathy: Role of Polyphenols. Front. Immunol..

[13] Luo M., Liu Z., Hu Z., He Q. (2022). Quercetin Improves Contrast-Induced Acute Kidney Injury through the HIF-1α/lncRNA NEAT1/HMGB1 Pathway. Pharm. Biol..

[14] Wang Y., Quan F., Cao Q., Lin Y., Yue C., Bi R., Cui X., Yang H., Yang Y., Birnbaumer L. (2021). Quercetin Alleviates Acute Kidney Injury by Inhibiting Ferroptosis. J. Adv. Res..

[15] Ahmed Z.S.O., Galal M.K., Drweesh E.A., Abou-El-Sherbini K.S., Elzahany E.A.M., Elnagar M.M., Yasin N.A.E. (2021). Protective Effect of Starch-Stabilized Selenium Nanoparticles against Melamine-Induced Hepato-Renal Toxicity in Male Albino Rats. Int. J. Biol. Macromol..

[16] Karunakar K.K., Edwin E.R., Gopalakrishnan M., Cheriyan B.V., Ramaiyan V., Karthikha V.S., Justin J.P. (2025). Advances in Nephroprotection: The Therapeutic Role of Selenium, Silver, and Gold Nanoparticles in Renal Health. Int. Urol. Nephrol..

[17] Al-Brakati A., Alsharif K.F., Alzahrani K.J., Kabrah S., Al-Amer O., Oyouni A.A., Habotta O.A., Lokman M.S., Bauomy A.A., Kassab R.B. (2021). Using Green Biosynthesized Lycopene-Coated Selenium Nanoparticles to Rescue Renal Damage in Glycerol-Induced Acute Kidney Injury in Rats. Int. J. Nanomed..

[18] Gligorić E., Vidić M., Teofilović B., Grujić-Letić N. (2025). Quercetin and Its Structural Analogs as NUDT5 Inhibitors: A Preliminary In Silico Study. Int. J. Mol. Sci..

[19] Hossain M.A., Fariha F.M., Hossain M.A., Kavey M.R.H., Shamim M., Hoque M.M., Hasan A.M.W., Rahman M.A., Harrath A.H., Rahman M.H. (2025). Binding Interaction and Stability Analysis of Quercetin and Its Derivatives as Potential Inhibitors of Triple Negative Breast Cancer (TNBC) against PARP1 Protein: An in-Silico Study. Curr. Pharm. Des..

[20] Gad E.S., Ashour A.M., Gad A.M., Khames A., Ibrahim S.G., Gadelmawla M.H.A., Mansour M. (2025). Hepatoprotection by Methylene Blue Against Doxorubicin Toxicity Through Coordinated Modulation of Oxidative Stress, ER Stress, and Apoptotic Pathways. Pharmaceuticals.

[21] Kim H., Shim H., Ranganath A., He S., Stevenson G., Allen J.E. (2024). Protein-Ligand Binding Affinity Prediction Using Multi-Instance Learning with Docking Structures. Front. Pharmacol..

[22] Gadelmawla M.H.A., Nasrallah H.H. (2025). The Protective Effects of Arbutin Against Colon Cancer: In Silico and in Vitro Studies. Sinai Int. Sci. J..

[23] Cruz-Rodriguez N., Tang H., Bateman B., Tang W., Deininger M. (2024). BCR::ABL1 Proteolysis-Targeting Chimeras (PROTACs): The New Frontier in the Treatment of Ph+ Leukemias?. Leukemia.

[24] Li Y., Wu Y., Gao S., Sun T., Jiang C. (2025). PROTAC Delivery in Tumor Immunotherapy: Where Are We and Where Are We Going?. J. Control. Release.

[25] Primikyri A., Chatziathanasiadou M.V., Karali E., Kostaras E., Mantzaris M.D., Hatzimichael E., Shin J.-S., Chi S.-W., Briasoulis E., Kolettas E. (2014). Direct Binding of Bcl-2 Family Proteins by Quercetin Triggers Its pro-Apoptotic Activity. ACS Chem. Biol..

[26] Najafi V., Yoosefian M., Hassani Z. (2023). Development of Venetoclax Performance Using Its New Derivatives on BCL-2 Protein Inhibition. Cell Biochem. Funct..

[27] Sadwal S., Bharati S., Dar Z.A., Kaur S. (2024). Chemopreventive Potential of Hydroethanolic *Murraya koenigii* Leaves Extract against DMBA Induced Breast Carcinogenesis: In-Silico and in-Vivo Study. J. Ethnopharmacol..

[28] Yu Z., Wei X., Dai L., Ma W., Li W., Li X., Han X.-L. (2025). Dual Binding Modes of Quercetin to BSA: Insights from Spectroscopy and Molecular Simulations in Amyloid Suppression. Spectrochim. Acta A Mol. Biomol. Spectrosc..

[29] Wu C.-M., Wu S.-C., Chung W.-J., Lin H.-C., Chen K.-T., Chen Y.-C., Hsu M.-F., Yang J.-M., Wang J.-P., Lin C.-N. (2007). Antiplatelet Effect and Selective Binding to Cyclooxygenase (COX) by Molecular Docking Analysis of Flavonoids and Lignans. Int. J. Mol. Sci..

[30] Mehallah H., Djebli N., Ngoc Khanh P., Xuan Ha N., Thi Ha V., Thu Huong T., Dinh Tung D., Manh Cuong N. (2024). In Silico and In Vivo Study of Anti-Inflammatory Activity of *Morinda longissima* (Rubiaceae) Extract and Phytochemicals for Treatment of Inflammation-Mediated Diseases. J. Ethnopharmacol..

[31] Zhang X.-Y., Xia K.-R., Wang Y.-N., Liu P., Shang E.-X., Liu C.-Y., Liu Y.-P., Qu D., Li W.-W., Duan J.-A. (2024). Unraveling the Pharmacodynamic Substances and Possible Mechanism of *Trichosanthis pericarpium* in the Treatment of Coronary Heart Disease Based on Plasma Pharmacochemistry, Network Pharmacology and Experimental Validation. J. Ethnopharmacol..

[32] Kellici T.F., Chatziathanasiadou M.V., Lee M.-S., Sayyad N., Geromichalou E.G., Vrettos E.I., Tsiailanis A.D., Chi S.-W., Geromichalos G.D., Mavromoustakos T. (2017). Rational Design and Structure-Activity Relationship Studies of Quercetin-Amino Acid Hybrids Targeting the Anti-Apoptotic Protein Bcl-xL. Org. Biomol. Chem..

[33] Wang J., Hu J., Qin D., Han D., Hu J. (2024). A Multi-Omics Mendelian Randomization Identifies Putatively Causal Genes and DNA Methylation Sites for Asthma. World Allergy Organ. J..

[34] Fatima I., Rehman A., Ding Y., Wang P., Meng Y., Rehman H.U., Warraich D.A., Wang Z., Feng L., Liao M. (2024). Breakthroughs in AI and Multi-Omics for Cancer Drug Discovery: A Review. Eur. J. Med. Chem..

[35] Wu M.-H., Xie Z., Zhi D. (2025). A Folding-Docking-Affinity Framework for Protein-Ligand Binding Affinity Prediction. Commun. Chem..

[36] Su T., Shen H., He M., Yang S., Gong X., Huang C., Guo L., Wang H., Feng S., Mi T. (2024). Quercetin Promotes the Proportion and Maturation of NK Cells by Binding to MYH9 and Improves Cognitive Functions in Aged Mice. Immun. Ageing.

[37] Khames A., Khalaf M.M., Gad A.M., Abd El-raouf O.M., Kandeil M.A. (2019). Nicorandil Combats Doxorubicin–Induced Nephrotoxicity via Amendment of TLR4/P38 MAPK/NFκ-B Signaling Pathway. Chem.-Biol. Interact..

[38] Jana S., Mitra P., Dutta A., Khatun A., Kumar Das T., Pradhan S., Kumar Nandi D., Roy S. (2023). Early Diagnostic Biomarkers for Acute Kidney Injury Using Cisplatin-Induced Nephrotoxicity in Rat Model. Curr. Res. Toxicol..

[39] Umar Ijaz M., Batool M., Batool A., Al-Ghanimd K.A., Zafar S., Ashraf A., Al-Misned F., Ahmed Z., Shahzadi S., Samad A. (2021). Protective Effects of Vitexin on Cadmium-Induced Renal Toxicity in Rats. Saudi J. Biol. Sci..

[40] Oh S.-M., Park G., Lee S.H., Seo C.-S., Shin H.-K., Oh D.-S. (2017). Assessing the Recovery from Prerenal and Renal Acute Kidney Injury after Treatment with Single Herbal Medicine via Activity of the Biomarkers HMGB1, NGAL and KIM-1 in Kidney Proximal Tubular Cells Treated by Cisplatin with Different Doses and Exposure Times. BMC Complement. Altern. Med..

[41] Wang Y., Gu Y., Gu X., Cooper D.B., Lewis D.F. (2023). Evidence of Kidney Injury in Preeclampsia: Increased Maternal and Urinary Levels of NGAL and KIM-1 and Their Enhanced Expression in Proximal Tubule Epithelial Cells. Front. Med..

[42] Friedmann Angeli J.P., Conrad M. (2018). Selenium and GPX4, a Vital Symbiosis. Free Radic. Biol. Med..

[43] Qing J., Zhang L., Fan R., Zhi H., Li C., Li Y., Wu J., Han C., Li Y. (2025). GPX4 Expression Changes in Proximal Tubule Cells Highlight the Role of Ferroptosis in IgAN. Sci. Rep..

[44] Khames A., Gad A.M., Abd El-raouf O.M., Kandeil M.A., Khalaf M.M. (2020). Sodium Thiosulphate Shows Promising Anti-Inflammatory Role against Doxorubicin-Induced Renal Injury Depending on Tlr4 Pathway Inhibition. Plant Arch..

[45] Mukherjee K., Chio T.I., Gu H., Sackett D.L., Bane S.L., Sever S. (2021). A Novel Fluorogenic Assay for the Detection of Nephrotoxin-Induced Oxidative Stress in Live Cells and Renal Tissue. ACS Sens..

[46] Wei L., Garces J.P.D., Kashani M., Dong Y., Kashani K.B. (2025). Role of the Complement System in Acute Kidney Injury: A Narrative Review. Mayo Clin. Proc..

[47] Li Z.-L., Li X.-Y., Zhou Y., Wang B., Lv L.-L., Liu B.-C. (2024). Renal Tubular Epithelial Cells Response to Injury in Acute Kidney Injury. eBioMedicine.

[48] Ren N., Wang W.-F., Zou L., Zhao Y.-L., Miao H., Zhao Y.-Y. (2023). The Nuclear Factor Kappa B Signaling Pathway Is a Master Regulator of Renal Fibrosis. Front. Pharmacol..

[49] Ortega L. (2010). Role of Cytokines in the Pathogenesis of Acute and Chronic Kidney Disease, Glomerulonephritis, and End-Stage Kidney Disease. Int. J. Interferon Cytokine Mediat. Res..

[50] Sindhughosa D.A., Pranamartha A. (2017). The Involvement of Proinflammatory Cytokines in Diabetic Nephropathy: Focus on Interleukin 1 (IL-1), Interleukin 6 (IL-6), and Tumor Necrosis Factor-Alpha (TNF-α) Signaling Mechanism. Bali Med. J..

[51] Ji Y., Zhao Z., Yang Y., Wang X., Qiao R., Yu X., Gong X., Feng Z., Hong Q. (2025). Mechanisms Underlying the Impact of Interleukin Family on Acute Kidney Injury: Pathogenesis, Progression, and Therapy. Research.

[52] Gao J., Deng Q., Yu J., Wang C., Wei W. (2024). Role of Renal Tubular Epithelial Cells and Macrophages in Cisplatin-Induced Acute Renal Injury. Life Sci..

[53] Sándor M., Kiss R., Keseru G.M. (2010). Virtual Fragment Docking by Glide: A Validation Study on 190 Protein-Fragment Complexes. J. Chem. Inf. Model..

[54] Shamsian S., Sokouti B., Dastmalchi S. (2024). Benchmarking Different Docking Protocols for Predicting the Binding Poses of Ligands Complexed with Cyclooxygenase Enzymes and Screening Chemical Libraries. Bioimpacts.

[55] Kaushik A., Parashar S., Ambasta R.K., Kumar P. (2024). Ubiquitin E3 Ligases Assisted Technologies in Protein Degradation: Sharing Pathways in Neurodegenerative Disorders and Cancer. Ageing Res. Rev..

[56] Gayathiri E., Prakash P., Kumaravel P., Jayaprakash J., Ragunathan M.G., Sankar S., Pandiaraj S., Thirumalaivasan N., Thiruvengadam M., Govindasamy R. (2023). Computational Approaches for Modeling and Structural Design of Biological Systems: A Comprehensive Review. Prog. Biophys. Mol. Biol..

[57] Alsubaie S., Merghani N., Abudawood M., Siddiqi N.J., Fatima S. (2025). Comparative Mechanistic Insights into Quercetin-Loaded Selenium Nanoparticles and Cisplatin in HCT116 Cells. ACS Omega.

[58] Lutfy R.H., Ashour A.M., Khames A., Elhemiely A.A., Alam-ElDein K.M., Faraag A.H.I., Hamed M.O.A., Abdel Daim Z.J., Attia N.I., Gadelmawla M.H.A. (2025). Targeting Oxidative Stress and Neuroinflammation: Epigallocatechin-3-Gallate-Selenium Nanoparticles Mitigate Sleep Deprivation-Induced Cortical Impairment. Int. J. Mol. Sci..

[59] Zhang Y., Du Y., Yu H., Zhou Y., Ge F. (2017). Protective Effects of *Ophiocordyceps lanpingensis* on Glycerol-Induced Acute Renal Failure in Mice. J. Immunol. Res..

[60] AlBasher G., Alfarraj S., Alarifi S., Alkhtani S., Almeer R., Alsultan N., Alharthi M., Alotibi N., Al-dbass A., Abdel Moneim A.E. (2020). Nephroprotective Role of Selenium Nanoparticles Against Glycerol-Induced Acute Kidney Injury in Rats. Biol. Trace Elem. Res..

[61] Cui Z., Zhao X., Amevor F.K., Du X., Wang Y., Li D., Shu G., Tian Y., Zhao X. (2022). Therapeutic Application of Quercetin in Aging-Related Diseases: SIRT1 as a Potential Mechanism. Front. Immunol..

[62] Backenroth R., Schuger L., Wald H., Popovtzer M.M. (1998). Glycerol-Induced Acute Renal Failure Attenuates Subsequent HgCl_2_-Associated Nephrotoxicity: Correlation of Renal Function and Morphology. Ren. Fail..

[63] Alrashdi B.M., Fehaid A., Kassab R.B., Rizk S., Habotta O.A., Abdel Moneim A.E. (2023). Biosynthesized Selenium Nanoparticles Using Epigallocatechin Gallate Protect against Pentylenetetrazole-Induced Acute Epileptic Seizures in Mice via Antioxidative, Anti-Inflammatory, and Anti-Apoptotic Activities. Biomedicines.

[64] Almeer R.S., AlBasher G.I., Alarifi S., Alkahtani S., Ali D., Abdel Moneim A.E. (2019). Royal Jelly Attenuates Cadmium-Induced Nephrotoxicity in Male Mice. Sci. Rep..

[65] Gounden V., Bhatt H., Jialal I. (2024). Renal Function Tests. StatPearls [Internet].

[66] Kristjansson R.P., Oddsson A., Helgason H., Sveinbjornsson G., Arnadottir G.A., Jensson B.O., Jonasdottir A., Jonasdottir A., Bragi Walters G., Sulem G. (2016). Common and Rare Variants Associating with Serum Levels of Creatine Kinase and Lactate Dehydrogenase. Nat. Commun..

[67] Callegari G.A., Novaes J.S., Neto G.R., Dias I., Garrido N.D., Dani C. (2017). Creatine Kinase and Lactate Dehydrogenase Responses after Different Resistance and Aerobic Exercise Protocols. J. Human Kinet..

[68] Ohkawa H., Ohishi N., Yagi K. (1979). Assay for Lipid Peroxides in Animal Tissues by Thiobarbituric Acid Reaction. Anal. Biochem..

[69] Green L.C., Wagner D.A., Glogowski J., Skipper P.L., Wishnok J.S., Tannenbaum S.R. (1982). Analysis of Nitrate, Nitrite, and [15N]Nitrate in Biological Fluids. Anal. Biochem..

[70] Ellman G.L., Courtney K.D., Andres V., Featherstone R.M. (1961). A New and Rapid Colorimetric Determination of Acetylcholinesterase Activity. Biochem. Pharmacol..

[71] Nishikimi M., Appaji Rao N., Yagi K. (1972). The Occurrence of Superoxide Anion in the Reaction of Reduced Phenazine Methosulfate and Molecular Oxygen. Biochem. Biophys. Res. Commun..

[72] Ciarlone A.E. (1978). Further Modification of a Fluorometric Method for Analyzing Brain Amines. Microchem. J..

[73] Gana K., Martin B., Canouet M.D. (2001). Worry and Anxiety: Is There a Causal Relationship?. Psychopathology.

[74] Arab H.H., Alsufyani S.E., Ashour A.M., Gad A.M., Elhemiely A.A., Gadelmawla M.H.A., Mahmoud M.A., Khames A. (2024). Targeting JAK2/STAT3, NLRP3/Caspase-1, and PK2/PKR2 Pathways with Arbutin Ameliorates Lead Acetate-Induced Testicular Injury in Rats. Pharmaceuticals.

[75] Komatsu R., Nakagawa M., Harada M., Tanaka Y. (1993). Changes in plasma and urinary calcium levels during cardiopulmonary bypass. Masui.

[76] Fang X., Yu L., Wang D., Chen Y., Wang Y., Wu Z., Liu R., Ren J., Tang W., Zhang C. (2020). Association Between SIRT1, Cytokines, and Metabolic Syndrome in Schizophrenia Patients With Olanzapine or Clozapine Monotherapy. Front. Psychiatry.

[77] Gadelmawla M.H.A., Alazzouni A.S., Farag A.H., Gabri M.S., Hassan B.N. (2022). Enhanced Effects of Ferulic Acid against the Harmful Side Effects of Chemotherapy in Colon Cancer: Docking and in Vivo Study. J. Basic Appl. Zool..

[78] Gaafar S.S., El Mekkawi A.R.O., Farag R.A., Gadelmawla M.H.A., Hussein A.M.H.M., Sayed M., Rayyan M., Basta D.G.A. (2025). Comparative Analysis of the Inflammatory Response of Human Gingival Fibroblasts to NeoSEALER Flo and CeraSeal Bioceramic Sealers: An in Vitro Study. BMC Oral Health.

[79] Alrashdi B.M., Ashry M., Germoush M.O., Fouda M., Abdel-Farid I., Massoud D., Shaldoum F., Abdel Moneim A.E., Gadel-Rab A.G., Mahrous M. (2025). Anti-Nephrotoxic, Antioxidant and Anti-Inflammatory Efficiency of Nigella Sativa Ethanolic Extract against CCl4-Induced Nephrotoxicity in Rats. Open Vet. J..

[80] Elhemiely A.A., El-Fayoumi S.H., Gadelmawla M.H.A., Mahran N.A., Gad A.M. (2025). Hesperidin Reduces Hepatic Injury Induced by Doxorubicin in Rat Model Through Its Antioxidative and Anti-Inflammatory Effects, Focusing on SIRT-1/NRF-2 Pathways. J. Biochem. Mol. Toxicol..

[81] Alrashdi B., Askar H., Germoush M., Fouda M., Abdel-Farid I., Massoud D., Alzwain S., Gadelmawla M., Ashry M. (2024). Evaluation of the Anti-Diabetic and Anti-Inflammatory Potentials of Curcumin Nanoparticle in Diabetic Rat Induced by Streptozotocin. Open Vet. J..

[82] El-Gneady F.F., Ashour A.M., Ashehri F.S., Khames A., Elhemiely A.A., Ahmed Mahmoud M., Mahran N.A., Alam-ElDein K.M., Gadelmawla M.H.A. (2025). Decoding the Effect of *Diceratella elliptica* on the Oxidative Stress–Inflammation Axis in Hyperthyroid-Induced Hepatotoxicity. J. Genet. Eng. Biotechnol..

[83] Alrashdi B.M., Askar H., Germoush M.O., Fouda M., Massoud D., Alzwain S., Abdelsater N., Salim L.M.S., Gadelmawla M.H.A., Ashry M. (2025). Cardioprotective, Anti-Inflammatory, and Antioxidative Outcome of Costus against Bleomycin-Induced Cardiotoxicity in Rat Model. J. Genet. Eng. Biotechnol..

[84] Ibrahim S.G., Abu-Dief A.M., Gad A.M., Gad E.S., Alzahrani A.Y.A., Alraih A.M., Barnawi I.O., Mansour M., Gadelmawla M.H.A., Khames A. (2025). Methylene Blue Mitigates Doxorubicin-Induced Cardiotoxicity via KEAP1/NRF2/GPX-4/Caspase3 Modulation. Int. J. Mol. Sci..

[85] Abdel-Wahhab K.G., Ashry M., Hassan L.K., El-Azma M.H., Elqattan G.M., Gadelmawla M.H.A., Mannaa F.A. (2024). Hepatic and Immune Modulatory Effectiveness of Lactoferrin Loaded Selenium Nanoparticles on Bleomycin Induced Hepatic Injury. Sci. Rep..

